# Radiolysis‐Driven Evolution of Gold Nanostructures – Model Verification by Scale Bridging In Situ Liquid‐Phase Transmission Electron Microscopy and X‐Ray Diffraction

**DOI:** 10.1002/advs.202202803

**Published:** 2022-07-03

**Authors:** Birk Fritsch, Tobias S. Zech, Mark P. Bruns, Andreas Körner, Saba Khadivianazar, Mingjian Wu, Neda Zargar Talebi, Sannakaisa Virtanen, Tobias Unruh, Michael P. M. Jank, Erdmann Spiecker, Andreas Hutzler

**Affiliations:** ^1^ Electron Devices (LEB) Department of Electrical, Electronic and Communication Engineering Friedrich‐Alexander‐Universität Erlangen‐Nürnberg Cauerstraße 6 91058 Erlangen Germany; ^2^ Institute of Micro‐ and Nanostructure Research (IMN) and Center for Nanoanalysis and Electron Microscopy (CENEM) Department of Materials Science and Engineering Friedrich‐Alexander‐Universität Erlangen‐Nürnberg Cauerstraße 3 91058 Erlangen Germany; ^3^ Institute for Crystallography and Structural Physics (ICSP) and Center for Nanoanalysis and Electron Microscopy (CENEM) Institute of Condensed Matter Physics Department of Physics Friedrich‐Alexander‐Universität Erlangen‐Nürnberg Staudtstraße 3 91058 Erlangen Germany; ^4^ Surface Science and Corrosion (LKO) Department of Materials Science and Engineering Friedrich‐Alexander‐Universität Erlangen‐Nürnberg Martensstraße 7 91058 Erlangen Germany; ^5^ Forschungszentrum Jülich GmbH Helmholtz Institute Erlangen‐Nürnberg for Renewable Energy (IEK‐11) Cauerstraße 1 91058 Erlangen Germany; ^6^ Fraunhofer Institute for Integrated Systems and Device Technology IISB Schottkystraße 10 91058 Erlangen Germany

**Keywords:** critical radius, gold nanoparticles, kinetic modelling, liquid‐phase transmission electron microscopy, oxidative etching, particle growth, radiolysis

## Abstract

Utilizing ionizing radiation for in situ studies in liquid media enables unique insights into nanostructure formation dynamics. As radiolysis interferes with observations, kinetic simulations are employed to understand and exploit beam‐liquid interactions. By introducing an intuitive tool to simulate arbitrary kinetic models for radiation chemistry, it is demonstrated that these models provide a holistic understanding of reaction mechanisms. This is shown for irradiated HAuCl_4_ solutions allowing for quantitative prediction and tailoring of redox processes in liquid‐phase transmission electron microscopy (LP‐TEM). Moreover, it is demonstrated that kinetic modeling of radiation chemistry is applicable to investigations utilizing X‐rays such as X‐ray diffraction (XRD). This emphasizes that beam‐sample interactions must be considered during XRD in liquid media and shows that reaction kinetics do not provide a threshold dose rate for gold nucleation relevant to LP‐TEM and XRD. Furthermore, it is unveiled that oxidative etching of gold nanoparticles depends on both, precursor concentration, and dose rate. This dependency is exploited to probe the electron beam‐induced shift in Gibbs free energy landscape by analyzing critical radii of gold nanoparticles.

## Graphical abstract

In situ investigations of liquid specimen via ionizing radiation provide unequaled information but suffer from radiolysis governing the observations. However, by suitable kinetic models these effects can be accounted for. This is herein demonstrated on HAuCl_4_‐solutions by comparison with scale‐bridging liquid‐phase transmission electron microscopy (LP‐TEM) and X‐ray diffraction (XRD) experiments. Moreover, a workflow is proposed to draw meaningful conclusions even under high dose rate conditions.

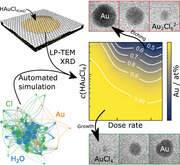

## 1 Introduction

Liquid‐phase transmission electron microscopy (LP‐TEM) emerged as a cutting‐edge in situ technique for investigation of processes at the nanoscale.^[^ ^1^, ^2^ ^]^ Particularly in the fields of catalysis,^[^ ^3^, ^4^ ^]^ energy materials and storage,^[^ ^5^, ^6^, ^7^ ^]^ soft‐matter studies,^[^ ^8^, ^9^ ^]^ or virology,^[^ ^10^, ^11^ ^]^ LP‐TEM has been enabling unprecedented insights into fundamental processes, such as nonclassical crystallization pathways^[^ ^12^, ^13^, ^14^ ^]^ and nanostructure self‐assembly.^[^ ^15^ ^]^

However, inherent inelastic electron‐matter interactions influence the Gibbs free energy landscape.^[^ ^16^, ^17^ ^]^ To enable LP‐TEM becoming a standard characterization method this needs to be accounted for appropriately. Besides electron‐beam induced heating,^[^ ^18^, ^19^ ^]^ radiolysis is a crucial factor during LP‐TEM,^[^ ^20^, ^21^, ^22^, ^23^ ^]^ even when radical scavengers are utilized^[^ ^24^, ^25^ ^]^ or radiolytic shielding via graphene membranes is employed.^[^ ^26^, ^27^ ^]^ Moreover, radiation chemistry has been identified as a prime driving force to study both reductive formation,^[^ ^28^, ^29^, ^30^ ^]^ and oxidative etching^[^ ^31^, ^32^, ^33^, ^34^, ^35^ ^]^ of metallic nanostructures. Operating between the poles of mitigation and utilization of radiation effects is not limited to LP‐TEM but is relevant for several techniques using ionizing radiation to investigate structures or processes in liquids in situ or *operando* such as X‐rays.^[^ ^36^ ^]^ Thus, understanding the radiation chemistry is *the* key for appropriate experimental design and interpretation.

However, most LP‐TEM studies solely rely on an incomplete description of the chemical environment, ignoring the intra‐ and interplay of complete clusters of chemical species. For example, investigations of gold nanoparticle evolution in aqueous tetrachloroauric acid (HAuCl_4_) solution, one of the most frequently used model systems in LP‐TEM,^[^ ^12^, ^28^, ^32^, ^37^, ^38^, ^39^, ^40^, ^41^, ^42^, ^43^, ^44^, ^45^, ^46^ ^]^ mainly rely on radiation chemistry of pure (and partially even deaerated) water.^[^ ^21^ ^]^ More accurate descriptions are sparse and only describe gold precursor reduction^[^ ^24^, ^38^ ^]^ or the radical chemistry of aqueous chloride solutions.^[^ ^37^ ^]^

In this study, we introduce an intuitive approach to translate chemical reaction sets into kinetic models, drastically simplifying and therefore allowing for a completion of the accurate description of complex solution chemistries during irradiation. We demonstrate the full potential of this approach by providing a comprehensive description of the radiation chemistry of aqueous HAuCl_4_ solutions, unveiling that the interplay between aqueous, gold‐, and chlorine‐containing species is crucial for an appropriate description of the solution chemistry. By using LP‐TEM and liquid cell X‐ray diffraction (XRD), we show that it is essential to consider the impact of ionizing radiation even for experiments with “low dose rate” irradiation techniques. Furthermore, we reveal how the initial HAuCl_4_ concentration determines the redox‐interplay during LP‐TEM and probe the impact on Gibbs free energy landscape by investigating the inherent stability of gold nanoparticles in oxidative environments.

## 2 Tool for Automated Radiolysis Simulations

Radiation chemistry inside a homogeneous volume element of a liquid phase irradiated with a steady electron beam is simulated under the assumption of an isotropic distribution of all contributing species. In this voxel, the chemical interplay is described by a set of coupled ordinary differential equations (ODEs),^[^ ^21^ ^]^ depicting the evolution of concentration *c* over time *t* of a reactant *i* depending on the concentrations of different reactants *l* and *n* (see Section S1.1 in the Supporting Information for details)

(1) $$\frac{\partial c_{i}}{\partial t}=\frac{\rho }{F}\psi G_{i}+\sum_{j}k_{j}\left(\prod_{l}c_{l}\right)-\sum_{m\neq j}k_{m}\left(\prod_{n}c_{n}\right)$$

Here, *ρ* denotes the liquid density, *F* the Faraday constant, *ψ* the dose rate in Gy s^−1^, *G_i_* the generation value (*g*‐value) of species *i*, and *k_j_, k_m_* the kinetic constant of reactions *j* and *m*. In a homogeneously irradiated liquid volume, a quasi‐closed system can emerge where diffusion of chemical species towards non‐irradiated volumes is negligible.^[^ ^32^, ^47^ ^]^

In the past, most radiolysis simulations have been achieved by manually creating the set of ordinary differential equations to solve Equation (1). This is a cumbersome and highly complicated process, prone to typing errors which are hard to detect. Consequently, extensions of the reaction set comprising pure water are sparse and mostly limited to comparatively few additional reactions. While proprietary tools are available to simulate reaction kinetics directly, they need to be adjusted to account for the generation of primary species which requires again a high level of computational skills and effort.

**Figure 1:**
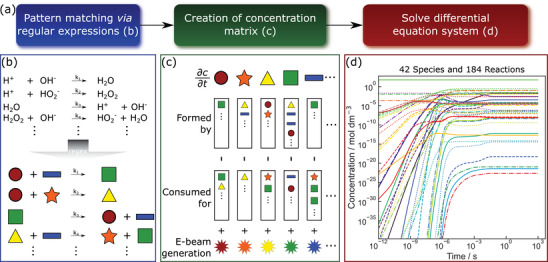
a) Workflow of the automated radiation‐chemistry simulation tool. b) Illustration of pattern matching using regular expressions (regex) for generating a c) set of coupled differential equations, which are then d) solved numerically.

To overcome these obstacles, we provide a routine solely relying on open‐source software. The code based on Python 3.7, NumPy,^[^ ^48^ ^]^ Matplotlib,^[^ ^49^ ^]^ pandas,^[^ ^50^ ^]^ and SciPy^[^ ^51^ ^]^ accepts a plain‐text file of the chemical reaction set as input and automatically generates the corresponding matrix of coupled differential equations. Its outcome has been validated against the MATLAB implementation of the reaction set of Schneider et al.,^[^ ^21^ ^]^ revealing excellent agreement in both temporal (Figure S1a, Supporting Information) and steady‐state evolution (Figure S1b, Supporting Information). The flowchart is sketched in **Figure** **1**a.

Figure 1b illustrates that this is achieved by using natural language processing via regular expressions (regex). Next, the reaction set is translated into a matrix to provide suitable input for a numerical ODE‐solver (Figure 1c).

An exemplary output of the solver is shown in Figure 1d for a comprehensive reaction set describing the evolvement and equilibration of relevant constituents in an irradiated aqueous HAuCl_4_ solution. It consists of 42 chemical species distributed over 184 reactions (see Supporting Information for details,^[^ ^21^, ^37^, ^38^, ^52^, ^53^, ^54^, ^55^, ^56^, ^57^, ^58^, ^59^, ^60^, ^61^, ^62^, ^63^, ^64^, ^65^, ^66^ ^]^ including an enlarged and annotated version of the plot depicted in Figure 1d (Figure S3, Supporting Information)). Figure S4 (Supporting Information) depicts the set based on graph theory,^[^ ^67^, ^68^ ^]^ revealing that the simulated steady‐state concentrations of the species do not necessarily coincide with their importance within the reaction set (c.f. Figure S5, Supporting Information). A tabular representation and further information can be found in the Supporting Information (Table S2 and Section S1 Radiolysis).

## 3 Results and Discussions

**Figure 2:**
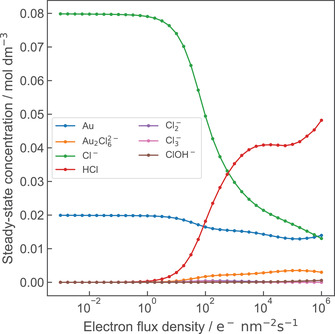
Nonaqueous species exceeding 1% of the initial HAuCl_4_ concentration of 20 × 10^−3^ m. A logarithmically‐scaled plot including aqueous species is given in Figure S7 in the Supporting Information.

**Figure** **2** displays steady‐state concentrations of the main nonaqueous radiolysis products of 20 × 10^−3^ m HAuCl_4_ solution. At dose rates relevant to LP‐TEM, steady‐state concentrations are approached within a few milliseconds, exemplarily shown in Figure 1d. Reactants are defined as a main product if their steady‐state concentration exceeds 1% of the initial HAuCl_4_ concentration at least once in the dose rate interval of irradiation. It is evident that not only a single but two Au‐containing species are numbered among this category, namely elementary Au and a gold dimer, Au_2_Cl_6_^2−^. The main amount of chlorine, in turn, is distributed over Cl^−^, molecular HCl, and Au_2_Cl_6_^2−^. The corresponding logarithmically‐scaled plot including aqueous species is displayed in Figure S7 in the Supporting Information.

Cl^−^ and Au appear to be the main products at low electron‐flux densities. HCl dominates the chlorine distribution at high electron‐flux densities, despite its strong dissociation behavior. Albeit most Au remains in a pristine state, a notable increase in the concentration of Au_2_Cl_6_^2−^ with ascending electron‐flux density is evident.

These outcomes are particularly relevant as their consequences are accessible experimentally:

- Gold ions will be reduced to elementary gold at low dose rates.
- HCl and Au_2_Cl_6_^2−^ exhibit high vapor pressures and are expected to appear in gaseous phases.
- Oxidative etching of gold nanostructures is fostered at high dose rates and high initial concentrations of HAuCl_4_.

These conclusions are evaluated in the following.

### 3.1 Dose Rate Requirements for Gold Nanostructure Growth

The most prominent consequence of irradiating HAuCl_4_‐solutions is the reduction of gold‐containing species to elementary Au, which agglomerates and forms crystalline structures easily detectable in TEM. For this mechanism, an electron‐flux density exceeding an estimated threshold between 2 × 10^3^ and 3 × 10^3^ e^−^ (nm^2^ s)^−1^ was reported previously.^[^ ^37^, ^38^ ^]^

However, our results of reaction‐kinetic simulations (Figure 2) do not indicate a threshold existing for irradiation of a 20 × 10^−3^ m HAuCl_4_ solution. This is remarkable, as previous models suggested such a phenomenon.^[^ ^38^ ^]^ Experiments demonstrated in Figure S8 (Supporting Information), display growth events of gold nanostructures at 540 e^−^ (nm^2^ s)^−1^. The growth rate decreased over time, suggesting the convergence against a steady‐state condition. As underlined by additional observations at 4.74 and 540 e^−^ (nm^2^ s)^−1^ (Figures S9 and S10, Supporting Information), this experiment indicates that the threshold stated above does not apply for our experiments.

**Figure 3:**
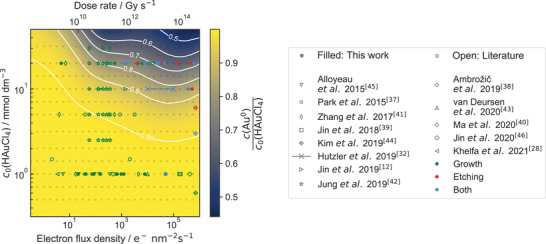
The equilibrium mole fraction of Au^0^ is mapped against electron‐flux density and initial concentration of HAuCl_4_. Gray open rectangles denote simulated values. The data in‐between is interpolated. Note that no extrapolation is performed. Level lines are shown in white. Green, red, and blue marks account for experimental observations relating to growth, etching, or both (simultaneously or subsequently). Unfilled marks annotate literature reports of radiolytic HAuCl_4_‐reduction in aqueous LP‐TEM experiments, while filled marks represent findings acquired during this work.

To further investigate this discrepancy, kinetic simulations have been performed for varying initial HAuCl_4_ concentrations and electron‐flux densities (**Figure** 3). Simulation results are indicated by a grid comprising of open gray rectangles. Parameters in‐between are interpolated cubically. The initial HAuCl_4_ concentration is the logical benchmark to characterize the system because it is not only an experimentally accessible parameter but also summarizes the initial pH value and the total amount of chlorine and gold present.

The resulting relative amount of gold reduced to Au^0^ is mapped in Figure 3. It is evident that the (almost) complete reduction of AuCl_4_^−^ to Au^0^ holds for all concentrations investigated at low dose rates and even at low initial HAuCl_4_ concentrations for all electron‐flux densities under investigation. Moreover, an overview of reports describing evolution of gold nanostructures from pristine HAuCl_4_ solutions in LP‐TEM is provided, showing good agreement with simulated mole fractions.^[^ ^28^, ^32^, ^37^, ^38^, ^39^, ^40^, ^41^, ^42^, ^43^, ^44^, ^45^, ^46^ ^]^

In this work, the dose rate is calculated for an electron energy of 300 keV and for a liquid film with a constant thickness of about 100 nm to describe our experiments performed in graphene‐supported microwell liquid cells (GSMLCs)^[^ ^47^, ^69^ ^]^ and other graphene‐based liquid cell architectures. Naturally, this cannot be matched perfectly with the broad range of experiments reported in literature. Therefore, the literature comparison shown cannot describe the gold ratio quantitatively but provides an orientation in terms of orders of magnitude, as variations induced by the experimental conditions, i.e. alterations in energy of the primary electrons and liquid thickness, are small compared to the regarded scales. Nonetheless, Figure 3 does not suggest a universal threshold. This is in accordance with the fact that gold nanostructure formation is regarded as a benchmark experiment for successfully sealed graphene‐based liquid cells.^[^ ^70^, ^71^ ^]^

To probe the (non)existence of an experimentally relevant threshold for gold precipitation in HAuCl_4_‐solution under exposure with ionizing radiation based on reaction kinetics, a 20 × 10^−3^ m HAuCl_4_‐solution was exposed to a Cu K_*α*_ X‐ray beam. With roughly 1 Gy s^−1^, this experiment was performed at a dose rate of about nine orders of magnitude below the lowest dose rate depicted in Figure 3.

**Figure 4:**
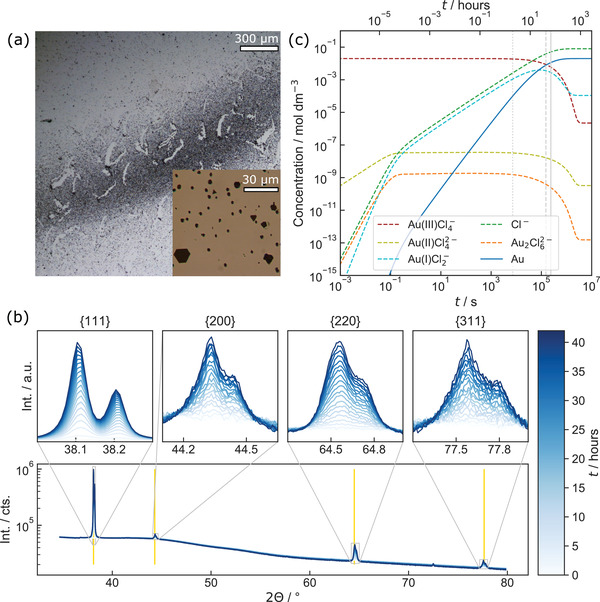
X‐ray irradiation of a 20 × 10^−3^ m HAuCl_4_‐solution. a) Optical images showing micrometer‐sized structures after 66 h of irradiation. The inset at higher resolution reveals that some form platelets of three‐ and sixfold symmetry, as expected for fcc‐crystals. An ex situ XRD scan reveals that they consist of gold (Figure S11b, Supporting Information). b) In situ XRD investigation for 42 h in 2 h intervals at a dose rate of roughly 1 Gy s^−1^. The vertical lines correspond to the lattice constant of gold.^[^ ^72^ ^]^ c) Corresponding kinetic simulation. For simplicity, only gold‐containing species and Cl^−^ are displayed. The grey lines mark the time after the first (2 h, dotted) and last in situ scan (42 h, dashed) experiment, as well as the time matching the experiment shown in (a) (66 h, solid).

Optical micrographs (**Figure** **4**a) reveal precipitation consisting of microstructures, which did not emerge in the corresponding reference experiment without X‐ray exposure. As shown in the inset, a notable amount of precipitation forms platelet structures with the typical three‐ or sixfold symmetry expected from fcc‐crystallites. Figure S11a (Supporting Information) shows that precipitates are visible by the naked eye. Lattice planes of the particle layer were investigated ex situ using XRD to characterize the crystal structures further. As demonstrated in Figure S11b (Supporting Information), the Bragg peaks match well to lattice planes of gold.^[^ ^72^ ^]^

Another ultra‐low dose rate‐mediated reductive gold synthesis was conducted for 42 h while repeatedly performing in situ XRD scans in 2 h intervals with a temporal resolution of 20 min (Figure 4b). An apparent increase in diffraction peak intensity with continuous irradiation is obtained. The visible peak splitting is due to incomplete monochromatization, which led to two diffraction peaks for *K*_*α*1_ and *K*_*α*2_, respectively. Additionally, due to the geometry of the liquid cell, the sample thickness could only be approximated roughly, leading to a slight shift in the peak baseline. Nevertheless, the obtained Bragg peaks match literature values for fcc gold.^[^ ^72^ ^]^ Notably, the first indication of peaks related to gold is visible even after the first measurement, indicating that irradiation‐induced gold reduction is not negligible during liquid‐phase X‐ray studies. This agrees with reports on X‐ray‐mediated gold reduction within minutes.^[^ ^73^ ^]^

Figure 4c shows a corresponding kinetic simulation. While *g*‐values precisely describing electron irradiation are available,^[^ ^21^ ^]^ more general *g‐*values acquired for low‐linear energy transfer (LET) radiation such as photons are used here.^[^ ^74^ ^]^ In contrast to kinetic simulations describing LP‐TEM conditions, a steady‐state condition is neither obtained within milliseconds nor within the duration of the experiment. Instead, a constant increase of Au^0^ over hours is expected until the system saturates in a steady state where almost all gold is reduced to pristine Au. As Au^0^ is insoluble in water, it is expected to precipitate immediately. Furthermore, surfaces providing nucleation sites or seed crystals are known to support this process (heterogeneous nucleation). Thus, this simulation well agrees with the constant increase in diffraction peak intensities presented in Figure 4b.

By exploiting the formation of Au^0^ as a marker for notable radiolysis, these observations demonstrate that this effect must be considered even when specimen are exposed to low dose rates. These findings are in‐line with further studies on gold reduction observed in photon‐mediated radiation chemistry caused by lasing,^[^ ^75^, ^76^ ^]^ UV exposure,^[^ ^77^ ^]^ or *γ*‐irradiation,^[^ ^54^, ^59^, ^60^, ^78^, ^79^, ^80^, ^81^ ^]^ mostly performed at dose rates many orders of magnitude lower than those utilized in synchrotron‐beam lines or typical LP‐TEM experiments.

Nevertheless, to explain threshold‐dose rates observed experimentally in LP‐TEM using silicon nitride‐windowed liquid cell architectures,^[^ ^37^, ^41^, ^42^ ^]^ different parameters apart from solution kinetics should be taken into account as well. Particularly, a considerable reservoir of non‐irradiated bulk‐like liquid^[^ ^82^ ^]^ or electron‐beam induced positive charging of Si_3_N_4_ membranes^[^ ^83^, ^84^, ^85^ ^]^ can cause gradients that may alter local concentrations. As shown in Figure 3, this can yield notably different mole fractions. Strictly speaking, this also applies to liquid cell architectures used for XRD‐studies. However, the volume ratio of irradiated liquid to the volume which is not exposed to the beam in this study is remarkably larger than similar ratios in typical commercial liquid cells for electron microscopy. Likewise, experiments conducted in liquid flow may exhibit phenomena fundamentally different to the results obtained in this study.

### 3.2 Volatile Species as Radiolysis Products

It has been reported previously that nanobubbles in LP‐TEM investigations of HAuCl_4_ solutions can etch gold nanostructures.^[^ ^32^ ^]^ It was concluded that this can only be related to reactive chlorine species within the gas phase, which can cause gold etching.^[^ ^86^ ^]^ The high concentration of HCl predicted by the kinetic simulations performed within this work agrees well with this observation, as HCl is known to be volatile. The formation of gaseous halide species has been reported for different ionizing radiations as well.^[^ ^87^ ^]^ It is noteworthy that this phenomenon appears regularly, as shown in Figure S12 in the Supporting Information. Here, a gold nanostructure is etched when directly contacting gas bubbles. If the gas bubble would mainly contain hydrogen, a passivation of the particle is expected to inhibit oxidative etching.^[^ ^33^ ^]^

In succession to etching, gold nanoparticles precipitate spatially isotropic in the vicinity of the gas bubbles (Figure S12b,c, Supporting Information).^[^ ^32^ ^]^ This is a strong indication of gaseous gold‐containing species, because only a gold‐transfer mechanism via the gaseous phase explains the isotropic appearance of the gold particles beneath the bubble.^[^ ^32^ ^]^ This fits well to the predicted formation of Au_2_Cl_6_^2−^, as such structures have already been reported to exist in gas phase previously.^[^ ^88^ ^]^ They could therefore cause such a nucleation behavior upon redissolution and supersaturation in the interfacing liquid.

### 3.3 Oxidative Etching of Gold Nanostructures

**Figure 5:**
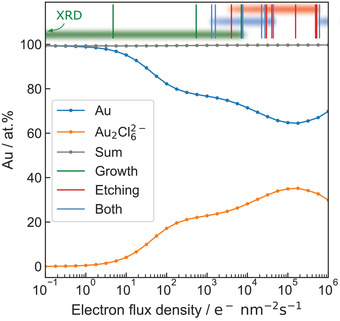
Steady‐state ratio of gold atoms distributed over the main radiolysis products Au^0^ (blue) and Au_2_Cl_6_^2−^ (orange) of a 20 × 10^−3^ m HAuCl_4_ solution plotted against electron‐flux density. Those two species store almost 100% of the gold atoms present (gray line). In addition, experiments performed within this work are marked with vertical lines and classified as growth (green), etching (red), or dynamics dominated by both processes (blue). The respective intervals are illustrated by shaded stripes.

At both high dose rates and high concentrations, Figure 3 reveals a dip in the relative Au^0^ steady‐state concentration, in‐line with the increase of Au_2_Cl_6_^2−^ concentration. For 20 × 10^−3^ m solutions, this is highlighted in **Figure** **5**. Here, the distribution of gold atoms within the main radiolysis products is again compared with morphological findings of the evaluated experiments.

Fifteen experimental observations were classified as either “growth” (green), “etching” (red), or “both” (blue), depending on the dominating phenomenon. While all growth events have been observed at low‐to‐medium electron‐flux densities, etching events occur more often at larger dose rates. Experiments observing both mechanisms have been identified at medium‐to‐high electron‐flux densities.

The trend appears to follow the concentration of Au^0^ shown in Figure 5. However, as the electron‐flux density intervals of all three cases overlap, the different electron‐flux density regimes must not be understood as strict boundaries but should be regarded as a likelihood to observe the respective phenomenon. Naturally, the statistical information of only fifteen observations is limited. Therefore, no in‐depth statistical analysis is performed on these interdependencies. Nevertheless, the trend indicated by the available data is in good agreement with the steady‐state conditions predicted by our simulations.

The decrease of pristine Au at high electron‐flux densities shown in Figures 3 and 5 appears only for (relatively) high initial HAuCl_4_ concentrations. This verifies that oxidative etching of gold itself is a function of the initial HAuCl_4_ concentration, as reported previously.^[^ ^44^ ^]^ As illustrated in Figure 3, this trend matches with our simulations, especially when a likely increase of concentration due to drying during the loading procedure is accounted for^[^ ^69^ ^]^ (not shown in Figure 3). Note that for the reported electron‐flux density (>10^7^ e^−^ nm^−^^2^ s^−1^), our simulation predicts the conversion of a substantial amount of solvent to H_2_, O_2_, and H_2_O_2_. As Kim et al.^[^ ^44^ ^]^ do not report any bubble formation, we assume that graphene membranes in the used graphene‐based liquid cells significantly mitigate radiolysis effects due to their electrical conductivity. Hence, these experiments are noted at slightly reduced electron‐flux densities in Figure 3.

The concentration dependency can be understood by the underlying chemistry—Gold reduction is mainly driven by solvated electrons (e_h_^−^) and H radicals^[^ ^54^ ^]^ which are primary radiolysis products in aqueous solutions and thus are independent on the initial HAuCl_4_ concentration. Gold oxidation, in turn, is suggested to occur via ClOH^−^ as an oxidant.^[^ ^37^, ^38^ ^]^ In contrast to primary radiolysis products, the formation of ClOH^−^ strongly depends on the number of chlorine atoms available in the system, which is determined by the concentration of HAuCl_4_.

At low HAuCl_4_ concentrations, the amount of ClOH^−^ is assumed to be insufficient to maintain a steady‐state in which a substantial net Au^0^ oxidation is notable. This hypothesis also agrees with studies showing a preferred gold etching in acidic environments at high (initial) chlorine concentrations^[^ ^34^, ^35^ ^]^ or demonstrating nucleation events at high dose rates, but low initial HAuCl_4_ concentrations.^[^ ^39^ ^]^ This is additionally demonstrated in Section S6 (Figure S18, Supporting Information).

In events combining growth and etching, these observations do not necessarily happen simultaneously but may show different temporal offsets, as shown in Figures S12 and S18 (Supporting Information), as well as literature reports.^[^ ^32^, ^44^, ^69^ ^]^ This can be understood when taking a closer look at the system itself. Our kinetic simulations assume a perfectly isotropic voxel, excluding phase boundaries or concentration gradients. Thus, steady‐state concentrations are reached within a fraction of a second and without any transient behaviors. However, concentration gradients, heterogeneous phase compositions, and resulting diffusion processes are expected to delay this process, shifting it to observable time scales and causing transients. In other words, only surface atoms of a nanostructure can contribute to a chemical reaction, whereas interior atoms remain passive.

**Figure 6:**
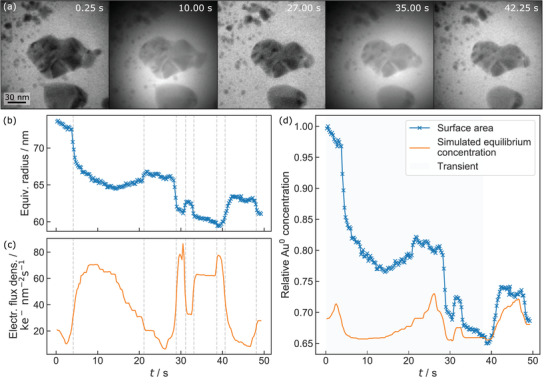
a) Series of bright‐field TEM micrographs showcasing the evolution of a gold nanostructure in 20 × 10^−3^ m HAuCl_4_ solution under repetitive in situ beam contraction. b) The evolution of the equivalent radius for the centered particle in (a) is directly related to the c) electron‐flux density. It is evident that the inflection points in (b) and (c) coincide. d) The relative steady‐state concentrations of Au and the relative evolution of the equivalent spherical surface area describing the centered particle in (a) is displayed.

Therefore, the system can be analyzed best at an experiment showing a slow response of the particles to mitigate the effect of steep overshoots or oscillations. **Figure** **6**a shows images of the temporal evolution of a gold nanostructure in LP‐TEM. The particle was present before electron irradiation and is thus regarded as a charge‐dissipation product caused by the graphene membrane (see Section S4 in the Supporting Information for details^[^ ^26^, ^27^, ^89^, ^90^, ^91^, ^92^ ^]^).

The nanostructure is exposed to repetitive beam contraction. Consequently, the dose rate is enhanced locally, altering the redox chemistry. By assuming a spherical geometry (see Section S5 in the Supporting Information), the particle size can be tracked by a single parameter,^[^ ^1^ ^]^ describing the sphere's radius with a cross sectional area equivalent to the projected particle size. This equivalent radius is plotted in Figure 6b. Its temporal evolution appears to be highly correlated with the applied electron‐flux density (Figure 6c). Their points of inflection coincide as indicated by the dotted lines. This has been observed reproducibly, as shown in Figures S16 and S17 in the Supporting Information. Thus, the gold reduction/oxidation process immediately responds to abrupt changes in local dose rate, in accordance with the rapid steady‐state formation in the kinetic simulations.

This is further investigated by translating the electron‐flux density to the amount of reduced gold by describing the electron‐flux density range of interest with a polynomial of third order (Figure S15, Supporting Information).

To compare this with experimental data, the equivalent spherical surface area with a cross‐section matching with the initial particle reflects the amount of Au^0^ available in the observed volume. Its initial value is assumed to correspond to the maximum value of gold surface atoms available because this would coincide with the irradiation at low electron‐flux densities.

As shown in Figure 6d, it is evident that simulation and measurement show a large offset that decays until about 38 s after which both plots converge (see shaded area in Figure 6d). The transients may cause the offset. In addition, the two curve shapes increase in similarity until they show consistent features. This is regarded as a strong indication of the quantitative validity of the simulated model.

This observation of transients which is already occurring within the irradiated area illustrates the significance of deliberate adjustments of the parameters relevant to the respective experimental set up. In particular, phenomena may be notably decelerated if large amounts of chemical species diffuse out of the irradiated area, as suggested previously.^[^ ^37^ ^]^ Consequently, we expect that by controlling the experimental parameters this growth delay is tailorable.

### 3.4 Implications of Dose Rate Dependency on Redox Chemistry and Gibbs Free Energy Landscape

By adjustment of experimental conditions in combination with fitting of the dose rate, the Gibbs free energy landscape can be tailored. This is herein probed by investigating the stability of gold nanoparticles.

As elucidated previously, classical nucleation theory does not fully describe nucleation of nanostructures because it does not cover so‐called nonclassical nucleation pathways like, e.g., formation via cluster clouds.^[^ ^12^, ^13^ ^]^ However, it provides reliable information about the minimum size at which a nanostructure can be regarded as stable. This is quantified by the critical radius *r*_crit_, which is reliably accessible during nanoparticle dissolution or etching.^[^ ^31^ ^]^ It is defined by the Gibbs free surface energy *γ*, the atomic volume *Ω*, and the difference in the chemical potential between the initial and the final phase under equilibrium conditions Δ*μ*.^[^ ^93^ ^]^

(2) $$r_{\mathrm{crit}}=\frac{2\gamma \Omega }{\Delta \mu }$$

Under similar experimental conditions, the thermal energy *k*_B_ *T* (with *k*_B_ being the Boltzmann constant) remains constant. Thus, Δ*μ* is governed by changes in substance (see Section S7 in the Supporting Information for details)^[^ ^93^, ^94^ ^]^

(3) $$\Delta \mu =k_{B}\;T\;\ln\left(\frac{c_{0}\left({\mathrm{HAuCl}}_{4}\right)}{c_{\mathrm{steady}\;\mathrm{state}}\left({\mathrm{Au}}^{0}\right)}\right)$$

**Figure 7:**
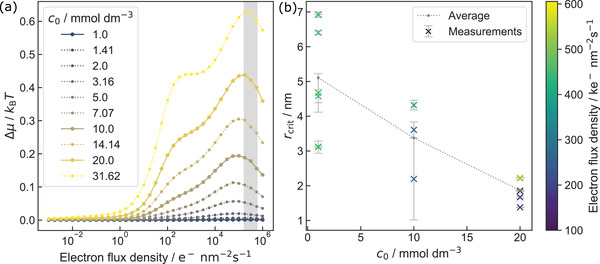
a) Chemical potential in units of *k*_B_ *T*, corresponding to the natural logarithm of the initial HAuCl_4_ concentration and the steady‐state concentration of Au^0^ as a function of electron‐flux density. The shaded area and solid lines relate to data relevant to b). b) *r*_crit_ for different initial concentrations of HAuCl_4_ solutions are shown. The gray‐shaded data correspond to the respective average of 5.1, 3.4, and 1.9 nm for 1, 10, and 20 × 10^−3^ m solutions of HAuCl_4_, respectively. The color bar depicts the electron‐flux density during the experiment. Error bars denote the precision of the least‐squares optimization (see Section S7 in the Supporting Information for details).

This natural logarithm is plotted in **Figure** **7**a as a function of electron‐flux density, showing an increase with *c*_0_ under high electron‐flux densities. According to Equation (2), this correlates with an expected decrease in *r*_crit_.

The described behavior was investigated by measuring *r*_crit_ of 17 different nanoparticles at three different initial concentrations of HAuCl_4_ (7 at 1 × 10^−3^ m, 3 at 10 × 10^−3^ m, and 7 at 20 × 10^−3^ m). *r*_crit_ was obtained by analyzing the slope of the etching rate (see Section S7 in the Supporting Information for details^[^ ^31^, ^95^, ^96^ ^]^). All experiments were conducted at electron‐flux densities of the same order of magnitude (1.6–6.1 × 10^5^_ _e^−^ (nm^2^ s)^−1^). As shown by the shaded region in Figure 7a, the effect of these variations on the chemical potential is small compared to the influence of *c*_0_. Resulting critical radii are plotted in Figure 7b depending on the initial HAuCl_4_ concentration.

Obtained critical radii scatter stronger at lower *c*_0_, emphasizing that *r*_crit_ is heavily affected by stochastic processes. Nevertheless, the reduction in scattering with *c*_0_ is an initial indicator for a dominating influence of *c*_0,_ which is even more pronounced when following the respective mean values. Here, a reduction of *r*_crit_ is observed with increasing initial chloride concentration, as predicted by relative changes in the radiation chemistry. The relative change amounting to a factor of two between 1 and 20 × 10^−3^ m HAuCl_4_ solutions is well matched by differences in Δ*μ* depicted in Figure 7a.

At 1 × 10^−3^ m, Figure 7a suggests no notable difference in chemical potential, as the steady‐state mol fraction of Au^0^ is expected to amount to unity here (Figure 3). Consequently, the observed transitions are expected to be reversible. Hence, the state can be interpreted as a dynamic equilibrium. This is in‐line with the experimental findings in Figure S18 (Supporting Information) showing reversible growth and dissolution. Furthermore, at 1 × 10^−3^ m the average value of *r*_crit_ is in the same range as values obtained for Pt‐nanoparticles in 0.228 m FeCl_3_ aqueous solution.^[^ ^31^ ^]^

The reversible process is still driven by the electron beam, because it defines the dynamic equilibrium condition itself (i.e., by changing the solution chemistry). However, this suggests that such dynamic equilibria could be well‐suited for analyzing material‐specific parameters.

Besides, a decrease in the Gibbs free surface energy *γ* would relate to a smaller *r*_crit_ (Equation (3)). Gold surfaces are, however, highly stabilized by chloride.^[^ ^97^ ^]^ Although the relative concentration of Cl^−^ decreases with the initial HAuCl_4_ concentration at the electron‐flux density range of interest, the absolute concentration still increases. Consequently, the gold surface is considered to remain saturated with Cl^−^ in all conducted experiments and, thus, changes in *γ* are expected to be negligible.

## 4 Conclusion

We introduce a comprehensive model for radiation chemistry in liquid for aqueous HAuCl_4_ solutions. This model shows good agreement with experimental findings using LP‐TEM, in situ XRD, and literature data. Our results emphasize that accurate and holistic modeling of beam effects is not only necessary but allows for drawing meaningful conclusions for unbiased (i.e. non‐irradiated) systems, even when operating at high dose‐rate regimes. Moreover, an automated open‐access tool to simulate kinetic models for radiation chemistry in liquid environments is provided which is applicable for arbitrary solutions and types of ionizing radiation.

## 5 Experimental Section

If not stated otherwise, LP‐TEM experiments were conducted using a GSMLC with a well depth of 100 nm and a Philipps CM30 (S)TEM in TEM mode and a frame rate of 4 frames per second. Liquid encapsulation was performed using 6–8 layer trivial‐transfer graphene (ACS Material) transferred onto holey carbon‐coated gold TEM grids (Quantifoil, PLANO). Fabrication and handling of GSMLCs have been elucidated elsewhere.^[^ ^47^, ^69^ ^]^ Experiments at 1 × 10^−3^ m HAuCl_4_ solution and electron‐flux densities below 10^4^ e^−^ (nm^2^ s)^−1^ were conducted using a Protochips Poseidon Select E‐Chip and a Thermo Fisher Scientific (formerly FEI) Titan^3^ Themis 300 (S)TEM. Both microscopes were operated at an acceleration voltage of 300 kV. All silicon nitride membranes were plasma‐treated prior to filling employing an ambient air plasma. Crystalline HAuCl_4_∙3 H_2_O (Alpha Aeasar) was dissolved in deionized water as a specimen solution.

XRD measurements were conducted with a Rigaku Smart‐Lab diffractometer using a Hypix 3000 solid‐state 2D detector. The Cu‐rotating anode X‐ray source was operated with an acceleration voltage of 45 kV and a current of 160 mA on the Wolfram filament. The X‐ray spectrum was not monochromatized to increase the primary beam intensity. 5° Soller slits were used on the incident and receiving optics. The incident slit was opened completely, resulting in a 7×15 mm beam. Both receiving slits were closed down to 0.2 mm. Measurements and irradiation were carried out in Bragg–Brentano geometry. The liquid cell containing aqueous 20 × 10^−3^ m HAuCl_4_ solution was lying flat in the goniometer center and was irradiated for about 1.75 h in between the in situ XRD scans. After the irradiation step, a 20 min *θ*/2*θ*‐scan from 35° to 80° was performed to record the developing Bragg peaks of nucleated gold particles. Irradiation and measurement were repeated 21 times, resulting in a total measurement time of 42 h.

### Data Analysis

Data curation of TEM experiments was performed using FIJI,^[^ ^98^ ^]^ as described elsewhere.^[^ ^69^ ^]^ All calculations were performed using Python, NumPy and the ScyPy framework.^[^ ^48^, ^51^ ^]^

## Conflict of Interest

The authors declare no conflict of interest.

## Authors Contribution

B.F.—Writing – original draft (lead), data curation (lead), Investigations (equal) – LP‐TEM at 20 × 10^−3^ m (lead), 1 × 10^−3^ m (supporting), formal analysis (lead), Software (lead) – Radiolysis tool (lead), GUI (supporting), Graph analysis (lead), Methodology (lead), Review and editing (lead). T.S.Z.—Investigations (supporting) – XRD‐Studies (lead), Formal analysis – (supporting), Writing – original draft (supporting), Review and editing (supporting). M.P.B.—Investigations (supporting) – Electrochemical studies (lead), Writing – original draft (supporting), Review and editing (supporting). A.K.—Software (supporting) – Graph analysis (supporting), Radiolysis tool (supporting) – code review, Validation (supporting), Formal analysis (supporting) – Graph analysis (supporting), Writing – original draft (supporting), Review and editing (supporting). S.K.—Software (supporting) – Radiolysis tool (supporting), Validation (supporting) – Verification against Matlab simulations (lead), Review and editing (supporting). M.W.—Investigations (supporting) – LP‐TEM at 1 × 10^−3^ m (equal), Review and editing (supporting). N.Z.T.—Software (supporting) – GUI (lead), Graph analysis (supporting), Review and editing (supporting). S.V.—Supervision (supporting), Review and editing (supporting), Project Administration (supporting), Funding Acquisition (supporting). T.U.—Supervision (supporting), Review and editing (supporting), Project Administration (supporting), Funding Acquisition (supporting). M.P.M.J.—Conceptualization (supporting), Supervision (equal), Review and editing (supporting), Project Administration (equal), Methodology (supporting), Funding Acquisition (supporting). E.S.—Conceptualization (supporting), Supervision (equal), Project Administration (equal), Methodology (supporting), Review and editing (supporting), Funding Acquisition (lead). A.H.—Investigations (equal) – LP‐TEM at 1 × 10 mm (equal), LP‐TEM at 10 × 10^−3^ m (lead), Data curation (supporting), Methodology (supporting), Conceptualization (lead), Review and editing (supporting), Supervision (equal), Project Administration (equal), Funding Acquisition (supporting).

## Supporting information

Supporting Information

Supplemental Video 1

Supplemental Video 2

Supplemental Video 3

Supplemental Video 4

Supplemental Video 5

## Data Availability

The data that support the findings of this study are available from the corresponding author upon reasonable request.
