# Supplementary material for: Radiolysis‐Driven Evolution of Gold Nanostructures – Model Verification by Scale Bridging In Situ Liquid‐Phase Transmission Electron Microscopy and X‐Ray Diffraction
Source: Adv Sci (Weinh). 2022 Jul 3;9(25):2202803. doi: 10.1002/advs.202202803 (PMC9443456; doi:10.1002/advs.202202803)
Supplement: Supplementary file 1 — Supporting Information [file ADVS-9-2202803-s006.pdf]

ADVANCED SCIENCE
Open Access

Supporting Information

for *Adv. Sci.*, DOI 10.1002/advs.202202803

Radiolysis-Driven Evolution of Gold Nanostructures – Model Verification by Scale Bridging In Situ Liquid-Phase Transmission Electron Microscopy and X-Ray Diffraction

Birk Fritsch, Tobias S. Zech, Mark P. Bruns, Andreas Körner, Saba Khadivianazar, Mingjian Wu, Neda Zargar Talebi, Sannakaisa Virtanen, Tobias Unruh, Michael P. M. Jank, Erdmann Spiecker and Andreas Hutzler**

Supporting Information

Radiolysis-Driven Evolution of Gold Nanostructures – Model Verification by Scale Bridging *in situ* Liquid-Phase Transmission Electron Microscopy and X-Ray Diffraction

Birk Fritsch, Tobias S. Zech, Mark P. Bruns, Andreas Körner, Saba Khadivianazar, Mingjian Wu, Neda Zargar Talebi, Sannakaisa Virtanen, Tobias Unruh, Michael P. M. Jank, Erdmann Spiecker, Andreas Hutzler**

*Corresponding authors: B.F. birk.fritsch@fau.de, A.H. a.hutzler@fz-juelich.de

S1 Radiolysis

S1.1 Concept

Radiation chemistry inside a homogeneous voxel of the liquid phase irradiated with a steady electron beam containing an isotropic distribution of all contributing species is simulated. Under homogeneous irradiation, a quasi-closed system can form within the irradiated volume with diffusion of chemical species towards non-irradiated volumes being negligible.[32,47] In this voxel, the chemical interplay is described by a set of coupled ordinary differential equations (ODEs). In the kinetic model, each ODE depicts the changes in concentration c over time t of a reactant i[21]:

$$\frac{\partial c_i}{\partial t} = \frac{\rho}{F}\psi G_i + \sum_j k_j \left(\prod_l c_l\right) - \sum_{m\neq j} k_m \left(\prod_n c_n\right) \qquad (S1)$$

In equation (S1), the first term relates to the physical interaction between the ionizing radiation and the liquid phase, with liquid density ρ, Faraday constant F, dose rate ψ, and generation value G_i, which can be approximated to zero for solvated species at small concentrations.[37] The generation values used in this work are listed in ***Table S1***. The second and third terms account for creation/consumption of species i during a chemical reaction with the rate constant k.

Table S1: List of generation values utilized within this work.

primary species	electron irradiation / (Molecules/100 eV)[21]	X-Ray irradiation / (Molecules/100 eV)[74]
e_h^-	3.47	2.60
H^+	4.42	3.10
OH^-	0.95	0.50
H_2O_2	0.47	0.70
H	1.00	0.66
OH	3.63	2.70
HO_2	0.08	0.02
H_2	0.17	0.45
H_2O	-5.68	-4.64

In the past, such simulations have been achieved by manually creating the set of ordinary differential equations to solve equation (S1). This is a cumbersome and highly complicated process, prone to hard-to-detect errors. Consequently, extensions of the reaction set comprising pure water are sparse and (mostly) limited to a few additional reactions.

A routine solely relying on open-source software is developed to overcome these limitations. The script based on Python 3.7, NumPy[48], Matplotlib[49], pandas[50], and SciPy[51] takes a plain-text file of the chemical reaction set as input and automatically creates the corresponding matrix of coupled differential equations. Its workflow is sketched in ***Figure 1***(a) of the main manuscript.

As illustrated in ***Figure 1***(b), this is achieved by using natural language processing via regular expressions (regex). Furthermore, the software is equipped with a tool to check the input reactions for consistent charge and atom balance. Next, the ODE matrix is translated into an

array to provide suitable input for the *solve_ivp* function of SciPy, where each row follows the syntax of equation (1). This is illustrated in ***Figure 1***(c).

By using the Fortran-based "*LSODA*" solver of SciPy, the solution of the coupled ODE-system is determined numerically. An exemplary output is shown ***Figure 1***(d) for the model described below, consisting of 42 different chemical species distributed over 184 reactions.

The electron flux density ϕ is used to account for experimentally accessible input variables. *Via* $\phi = n\ e\ /\ (A_{\phi} t)$, with the elementary charge e, the number of electrons n per time interval t (here: 1 s), and area A_{ϕ} (here: 1 nm²), it is converted into the dose rate experienced during the experiment:[18]

$$\psi = \left(1 + \frac{d}{\lambda}\right)\frac{S}{e}\phi \qquad (S2)$$

with d as liquid thickness ($d \leq 100$ nm in this study), λ as inelastic mean free path (380 nm at 300 kV acceleration voltage[52]), and S as stopping power (2.36 MeV(cm)²g^{-1} at 300 keV electrons in water[53]).

S1.2 Validation

The tool has been validated against the MATLAB implementation of the reaction set of Schneider *et al*.,[21] and shows excellent agreement in both temporal (***Figure S1***(a)) and steady-state evolution (***Figure S1***(b)).

The code is available at GitHub at https://github.com/BirkFritsch. It is held in a modular fashion to simplify possible extensions and incorporations into third-party software. The code can be easily executed using Anaconda, or any other Python environment with the required third-party modules installed. A list of the dependencies is given on GitHub.

Furthermore, it is extended by a graphical user interface (GUI) allowing for simulation of reaction kinetics without any programming knowledge (***Figure S2***). The GUI is also available on GitHub. The tool incorporates an option to check for errors in the reaction text file by

verification of mass- and charge balance for each individual reaction and a scan for doublings. Doublings are reactions where an identical set of educts yields an identical set of products. Furthermore, the tool offers the option to verify that mass was conserved during the kinetic simulation. This differs significantly from the mass balance check of the reaction text file, because here, also the generation value balance is crucial. ***Figure S6*** provides an exemplary output for the reaction set described below.

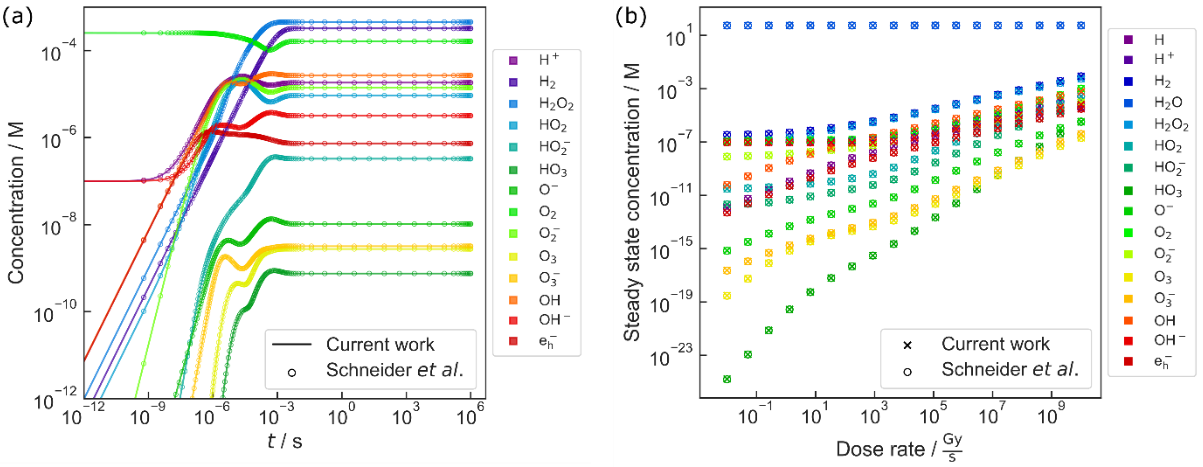

Figure S1: Comparison of water radiolysis simulation using the tool presented here and the MATLAB-script published by Schneider *et al.*[21]. (a) Temporal simulation at 20 MGy s^{-1} showing only the radiolysis products. (b) Steady-state concentrations including the solvent concentration.

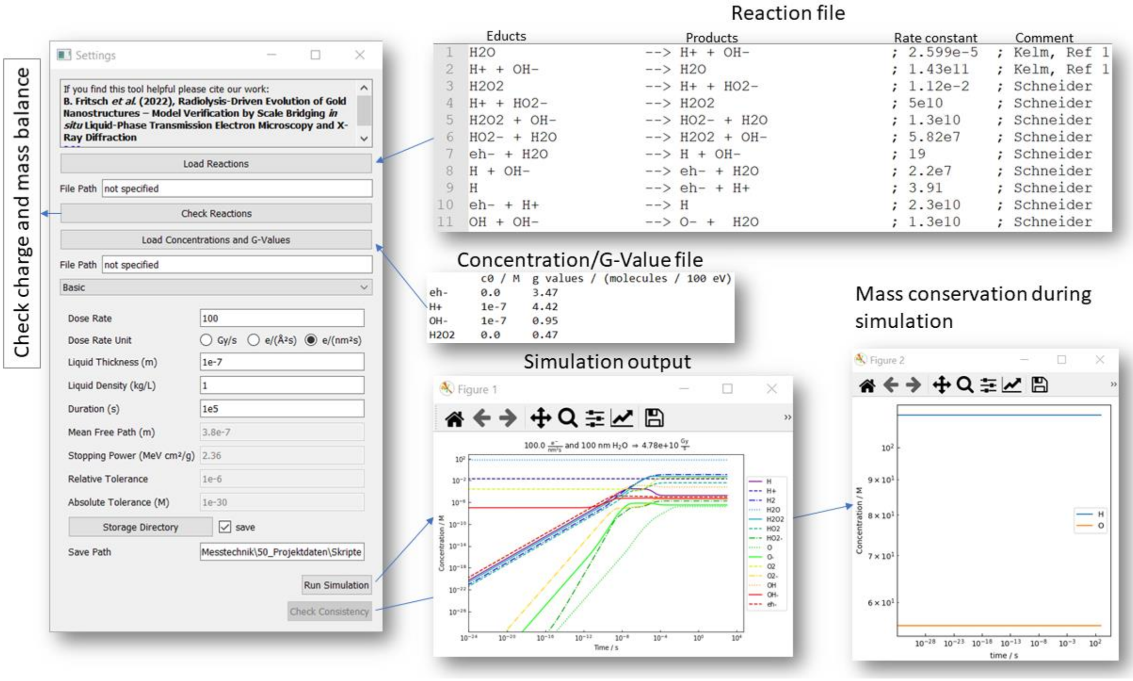

Figure S2: Screenshot of the input text files, the GUI window, and its main functionalities.

The reaction set consists of three columns separated by a semicolon ";" (see **Figure S2** for an example of first eleven reactions used in this work):

- The left-hand side is the most important part, as it describes the chemical reaction:
  - Educts and products are separated by " -->".
  - Arbitrary names for agents can be used, if **they are used consistently throughout the whole set**. This in turn also means that **every name must be unique**. Note that H* and H would be two different species! **Extra attention has to be paid when copying content from another source**, e.g. a pdf file, etc.. Only because symbols look similar, they are not necessarily the same ASCII symbol. A major issue is "-" and "–".
    - WARNING: While the kinetic simulation itself does not require meaningful names; the error handling (partially) requires a chemistry-like notation. Hence, we suggest using the following convention that leans on the usual nomenclature for chemical formulae:
      - Every chemical element starts with a capital letter, e.g. H, Au.
      - Indices denoting the amount of an element in a chemical formula are written as Arabic numbers in-line. (e.g. H2O, Cl2, Au), whereas an index of "1" can be omitted.
      - Charges are written at the end of a sum formula. In case of a single charge, a sole "+" or "-" suffices. If more charges are present, this should be indicated by a "^" followed by an Arabic number (e.g. Cl-, H+, Au2Cl6^2- for Cl^-, H^+, or $Au_2Cl_6^{2-}$).
      - Parentheses indicating oxidation states are fine (e.g. Au(III)Cl4-).
  - Stoichiometric factors are written in front of the reactant separated by a space " ". E.g. **2** H2O.
  - Agents and their stoichiometric factors are separated by " + ". Take care to put the space in front and after the "+" so that the code recognizes it as an operator.
- The right-hand side is providing the reaction rate constants in mol^{-n+1} dm$^{3(n-1)}$ s^{-1} with n as reaction order. You can abbreviate large numbers by using scientific notation e.g. 5e9 (= $5 \cdot 10^9$) instead of 5000000000, or 1.3e-1 instead of 0.13.
  - It is important, however, to **use "." as decimal spacer** and do not type any spacer symbols such as " " or ",".
  - **Do not type a unit** here, just provide a number.
- These would be **valid lines** in the code:
  - 2 eh- + 2 H2O --> H2 + 2 OH- ; 5.5e9 ; a comment.
  - H+ + eh- --> H ; 0 ; just another comment.
- **Do not leave blank lines** in between the reaction set.
- **Do not write headings** in the reaction set.
- **Store** the reaction set as ".txt" file, e.g. "reactions.txt".

The second file is related to the initial concentrations and G-values of the species if known (see again **Figure S2** for an example of first four radiolytic species used in this work):

- Here, you can enter a starting concentration in M and a constant G-value if required.
- **It is not necessary to list all species here**. If it is not listed, the program displays a warning and automatically sets both, *G* value and the initial concentration of this

species to 0. Again, pay attention to use the **identical syntax** for your agents in the file as in the reactions.

- The file consists of **three columns separated by a tab stop**.
    - The first one lists the species name identical to the used one in the reaction set file, the second one the initial concentration in M, and the third one the G-value in molecules/100eV.
- Do not comment in this file.
- Do not leave blank lines in between.

Store the reaction set as ".txt" file, e.g. "concentrations.txt".

When creating a reaction set it is important to ensure that every species included exhibits at least one formation and one decay pathway. If the former is missing, it will not be formed. In the latter case, its concentration will inevitably rise until one precursor species is exhausted. However, as redox reactions are in principle reversible such a simulation is usually meaningless if the counter reaction is not regarded.

In our experience, it is possible to set up a simulation for a novel reaction set within short notice, if all required reactions and parameters are known. As elucidated by Schneider *et al.*,[21] the NIST solution kinetics database (https://kinetics.nist.gov/solution/) provides a good starting point to survey possible radiation-chemistry reactions.

A more comprehensive manual of the GUI and working examples including the $HAuCl_4$ set used in this work (see ***Table S2*** below) can be found on GitHub.

S1.3 Kinetic modeling of $HAuCl_4$ solutions

During this work, a comprehensive reaction set for $HAuCl_4$ irradiation consisting of 184 reactions and 42 reactive species has been created, based on the set proposed by Schneider *et al.*[21]. On top, aqueous species are appended by the O-radical and O_4 to account for their importance in reactions with chlorine species and the spontaneous decomposition of H_2O_2 and O_3. The interaction of gold with radiolytic species is based on Dey *et al.*[54] and Ambrožič *et al.*[38]. The chlorine reactions mainly stem from combining the works of Kläning, Sehested, and Holcman[56], Kelm and Bohnert[57], and Levanov and Iskaikina[58], while the amount of chloride ligands in the gold species proposed Dey *et al.*[54]**,** and Buxton and Sellers[59] is following

Gachard *et al.*[60]. In accordance with Park *et al.*[37], $HAuCl_4$ was assumed to be fully deprotonated prior to irradiation. As shown in ***Figure S3***, steady states are formed within a fraction of a second at dose rates relevant to TEM.

A graph representation of the reaction set is shown in ***Figure S4***. In graph theory, every reactant can be represented by a node. The links (arrows) connecting the nodes represent every chemical reaction where the reactant is involved. An arrow spanning from reactant A to reactant B is to be read as '*A is an educt forming B*'. Thus, for a schematic reaction A + B → C + D, four arrows would be drawn, connecting A with C, A with D, B with C, and B with D. This concept is illustrated in the legend of ***Figure S4***. Additionally, the weight of every link represents the decadic logarithm of the respective kinetic constant times the stoichiometric factor of the educt node, contributing to the width of the link, as suggested elsewhere.[68]

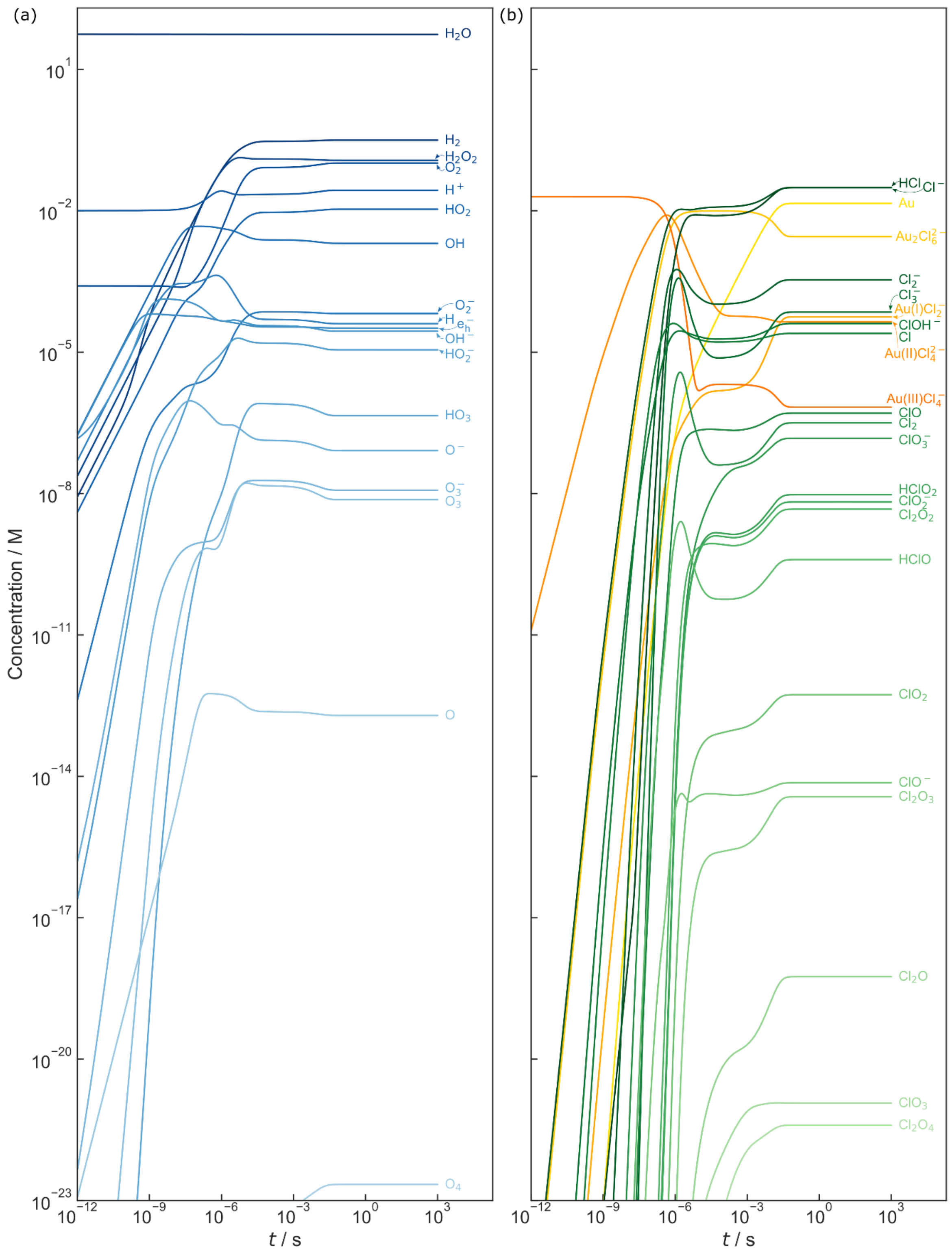

Figure S3: Temporal evolution of the concentration of (a) water-based and (b) gold/chlorine-containing species of irradiation of 20 mM $HAuCl_4$ at 1000 $e^-(nm^2 s)^{-1}$.

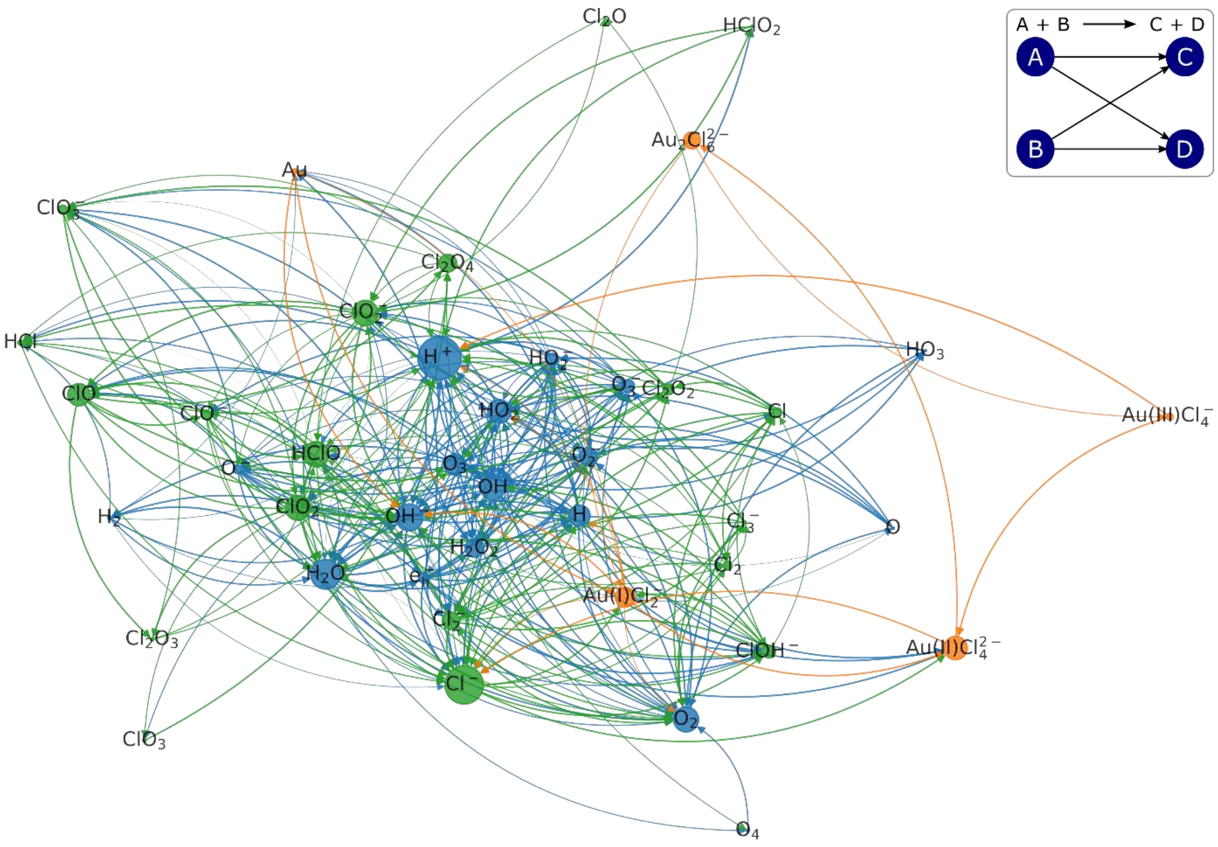

Figure S4: Graph representation of the utilized reaction set.

To account for the relevance of the respective reactant within the reaction set, the size of a node is representing its betweenness centrality, a measure corresponding to the direct connections of different species traversing this node. The color of a link matches the color of the node where it originates from. Graphs are created via NetworkX.[67] To account for the generation of primary species, they are accounted for by pseudo-reactions with a pseudo-reaction constant of $G_i \cdot 10^{12}$ s^{-1}.

To analyze the relevance of a species within the reaction set, the harmonic centrality H is used. It can be understood as the amount of information traversing a node within a network with weighted links. Transferred to reaction chemistry, the ‘information’ relates to the individual structures being changed. Calculating the difference ΔH of incoming and outgoing paths (ΔH) is feasible, because the network is directed, i.e. links are not bi-directional.

$$\Delta H = H_{incoming} - H_{outgoing} \qquad (S3)$$

Positive values of ΔH describe more 'chemical information' (structures forming the species node) is being transferred to the species than carried away (other species formed *via* the species node) and *vice versa*.

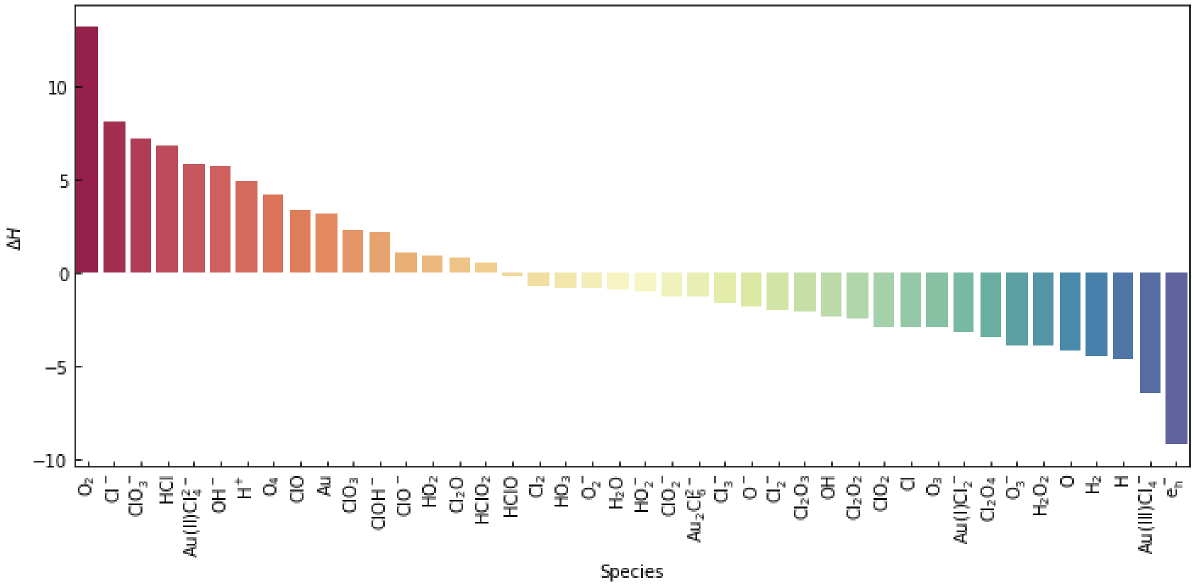

Figure S5: Visualization of the difference in harmonic centrality of in- and outgoing links.

Unsurprisingly, e_h^- is the species with the lowest value of ΔH, meaning it is involved in many more reactions that consume than yield solvated electrons. O_2 and Cl^- are ranked highest, i.e. fast reactions appear to dominate its formation in comparison to reactions depleting the species' concentration. Interestingly, H_2 and H_2O_2 show a negative ΔH, although high concentrations are predicted by the steady states of the kinetic simulations. The latter are assumed to be attributed to the primary generation rate during radiolysis. This can be vastly underrepresented by the introduced pseudo-reactions, depending on the dose rate.

Moreover, it must be borne in mind that the graph analysis shown here does not take differences in concentration into account but only analyzes the network structure itself. Therefore, species can show node sizes in ***Figure S4*** or ranks in ***Figure S5*** that do not agree with the steady state concentrations simulated. Thus, graph analysis cannot substitute proper kinetic simulation to

predict actual chemical conditions during LP-TEM. Instead, it provides an understanding of the underlying connections independent of dose rate and initial concentrations.

A tabular representation of the kinetic model can be found below (***Table S2***). The model ensures mass conservation during the temporal evolution in the simulation (***Figure S6***).

Table S2: Kinetic model to describe radiolysis of aqueous $HAuCl_4$ solutions. Here, *k* denotes the respective kinetic constant. Its unit amounts to mol^{-n+1} dm$^{3(n-1)}$ s^{-1} with *n* as reaction order.

	Reaction			*k*	Source
1	H_2O	→	$H^+ + OH^-$	$2.599 \cdot 10^{-5}$	[57]
2	$H^+ + OH^-$	→	H_2O	$1.43 \cdot 10^{11}$	[57]
3	H_2O_2	→	$H^+ + HO_2^-$	$1.119 \cdot 10^{-1}$	[21]
4	$H^+ + HO_2^-$	→	H_2O_2	$5 \cdot 10^{10}$	[21]
5	$H_2O_2 + OH^-$	→	$HO_2^- + H_2O$	$1.3 \cdot 10^{10}$	[21]
6	$HO_2^- + H_2O$	→	$H_2O_2 + OH^-$	$5.82 \cdot 10^{7}$	[21]
7	$e_h^- + H_2O$	→	$H + OH^-$	$1.9 \cdot 10^{1}$	[21]
8	$H + OH^-$	→	$e_h^- + H_2O$	$2.2 \cdot 10^{7}$	[21]
9	H	→	$e_h^- + H^+$	$3.9 \cdot 10^{0}$	[21]
10	$e_h^- + H^+$	→	H	$2.3 \cdot 10^{10}$	[21]
11	$OH + OH^-$	→	$O^- + H_2O$	$1.3 \cdot 10^{10}$	[21]
12	$O^- + H_2O$	→	$OH + OH^-$	$1 \cdot 10^{8}$	[21]
13	OH	→	$O^- + H^+$	$1.259 \cdot 10^{-1}$	[21]
14	$O^- + H^+$	→	OH	$1 \cdot 10^{11}$	[21]
15	HO_2	→	$O_2^- + H^+$	$1.346 \cdot 10^{6}$	[21]
16	$O_2^- + H^+$	→	HO_2	$5 \cdot 10^{10}$	[21]
17	$HO_2 + OH^-$	→	$O_2^- + H_2O$	$5 \cdot 10^{10}$	[21]
18	$O_2^- + H_2O$	→	$HO_2 + OH^-$	$1.862 \cdot 10^{1}$	[21]
19	$e_h^- + OH$	→	OH^-	$3 \cdot 10^{10}$	[21]
20	$e_h^- + H_2O_2$	→	$OH + OH^-$	$1.1 \cdot 10^{10}$	[21]
21	$e_h^- + O_2^- + H_2O$	→	$HO_2^- + OH^-$	$1.3 \cdot 10^{10}$	[21]
22	$e_h^- + HO_2$	→	HO_2^-	$2 \cdot 10^{10}$	[21]
23	$e_h^- + O_2$	→	O_2^-	$1.9 \cdot 10^{10}$	[21]
24	$2\ e_h^- + 2\ H_2O$	→	$H_2 + 2\ OH^-$	$5.5 \cdot 10^{9}$	[21]
25	$e_h^- + H + H_2O$	→	$H_2 + OH^-$	$2.5 \cdot 10^{10}$	[21]
26	$e_h^- + HO_2^-$	→	$O^- + OH^-$	$3.5 \cdot 10^{9}$	[21]
27	$e_h^- + O^- + H_2O$	→	$OH^- + OH^-$	$2.2 \cdot 10^{10}$	[21]
28	$e_h^- + O_3^- + H_2O$	→	$O_2 + OH^- + OH^-$	$1.6 \cdot 10^{10}$	[21]

29	$e_h^- + O_3$	→	O_3^-	$3.6 \cdot 10^{10}$	[21]
30	$H + H_2O$	→	$H_2 + OH$	$1.1 \cdot 10^{1}$	[21]
31	$H + O^-$	→	OH^-	$1 \cdot 10^{10}$	[21]
32	$H + HO_2^-$	→	$OH + OH^-$	$9 \cdot 10^{7}$	[21]
33	$H + O_3^-$	→	$OH^- + O_2$	$1 \cdot 10^{10}$	[21]
34	$2\ H$	→	H_2	$7.8 \cdot 10^{9}$	[21]
35	$H + OH$	→	H_2O	$7 \cdot 10^{9}$	[21]
36	$H + H_2O_2$	→	$OH + H_2O$	$9 \cdot 10^{7}$	[21]
37	$H + O_2$	→	HO_2	$2.1 \cdot 10^{10}$	[21]
38	$H + HO_2$	→	H_2O_2	$1.8 \cdot 10^{10}$	[21]
39	$H + O_2^-$	→	HO_2^-	$1.8 \cdot 10^{10}$	[21]
40	$H + O_3$	→	HO_3	$3.8 \cdot 10^{10}$	[21]
41	$2\ OH$	→	H_2O_2	$3.6 \cdot 10^{9}$	[21]
42	$OH + HO_2$	→	$H_2O + O_2$	$6 \cdot 10^{9}$	[21]
43	$OH + O_2^-$	→	$OH^- + O_2$	$8.2 \cdot 10^{9}$	[21]
44	$OH + H_2$	→	$H + H_2O$	$4.3 \cdot 10^{7}$	[21]
45	$OH + H_2O_2$	→	$HO_2 + H_2O$	$2.7 \cdot 10^{7}$	[21]
46	$OH + O^-$	→	HO_2^-	$2.5 \cdot 10^{10}$	[21]
47	$OH + HO_2^-$	→	$HO_2 + OH^-$	$7.5 \cdot 10^{9}$	[21]
48	$OH + O_3^-$	→	$O_3 + OH^-$	$2.6 \cdot 10^{9}$	[21]
49	$OH + O_3^-$	→	$2\ O_2^- + H^+$	$6 \cdot 10^{9}$	[21]
50	$OH + O_3$	→	$HO_2 + O_2$	$1.1 \cdot 10^{8}$	[21]
51	$HO_2 + O_2^-$	→	$HO_2^- + O_2$	$8 \cdot 10^{7}$	[21]
52	$HO_2 + HO_2$	→	$H_2O_2 + O_2$	$7 \cdot 10^{5}$	[21]
53	$HO_2 + O^-$	→	$O_2 + OH^-$	$6 \cdot 10^{9}$	[21]
54	$HO_2 + H_2O_2$	→	$OH + O_2 + H_2O$	$5 \cdot 10^{-1}$	[21]
55	$HO_2 + HO_2^-$	→	$OH + O_2 + OH^-$	$5 \cdot 10^{-1}$	[21]
56	$HO_2 + O_3^-$	→	$O_2 + O_2 + OH^-$	$6 \cdot 10^{9}$	[21]
57	$HO_2 + O_3$	→	$HO_3 + O_2$	$5 \cdot 10^{8}$	[21]
58	$2\ O_2^- + 2\ H_2O$	→	$H_2O_2 + O_2 + 2\ OH^-$	$1 \cdot 10^{2}$	[21]
59	$O_2^- + O^- + H_2O$	→	$O_2 + 2\ OH^-$	$6 \cdot 10^{8}$	[21]
60	$O_2^- + H_2O_2$	→	$OH + O_2 + OH^-$	$1.3 \cdot 10^{-1}$	[21]
61	$O_2^- + HO_2^-$	→	$O^- + O_2 + OH^-$	$1.3 \cdot 10^{-1}$	[21]
62	$O_2^- + O_3^- + H_2O$	→	$O_2 + O_2 + 2\ OH^-$	$1 \cdot 10^{4}$	[21]
63	$O_2^- + O_3$	→	$O_3^- + O_2$	$1.5 \cdot 10^{9}$	[21]
64	$2\ O^- + H_2O$	→	$HO_2^- + OH^-$	$1 \cdot 10^{9}$	[21]
65	$O^- + O_2$	→	O_3^-	$3.6 \cdot 10^{9}$	[21]

66	$O^- + H_2$	→	$H + OH^-$	$8 \cdot 10^7$	[21]
67	$O^- + H_2O_2$	→	$O_2^- + H_2O$	$5 \cdot 10^8$	[21]
68	$O^- + HO_2^-$	→	$O_2^- + OH^-$	$4 \cdot 10^8$	[21]
69	$O^- + O_3^-$	→	$O_2^- + O_2^-$	$7 \cdot 10^8$	[21]
70	$O^- + O_3$	→	$O_2^- + O_2$	$5 \cdot 10^9$	[21]
71	O_3^-	→	$O_2 + O^-$	$3.3 \cdot 10^3$	[21]
72	$O_3^- + H^+$	→	$O_2 + OH$	$9 \cdot 10^{10}$	[21]
73	HO_3	→	$O_2 + OH$	$1.1 \cdot 10^5$	[21]
74	H_2O_2	→	$H_2O + O$	$1 \cdot 10^{-3}$	[57]
75	$2\ O$	→	O_2	$1 \cdot 10^9$	[57]
76	O_3	→	$O_2 + O$	$3 \cdot 10^{-6}$	[61]
77	$2\ O_3^- + H_2O$	→	$OH^- + HO_2^- + 2\ O_2$	$1 \cdot 10^4$	[58]
78	$2\ HO_3$	→	$H_2O_2 + 2\ O_2$	$5 \cdot 10^9$	[58]
79	$O_3 + OH^-$	→	$HO_2^- + O_2$	$1 \cdot 10^2$	[58]
80	$O_2 + O$	→	O_3	$4 \cdot 10^9$	[55]
81	$H_2O_2 + O$	→	$OH + HO_2$	$1.6 \cdot 10^9$	[62]
82	$O + HO_2^-$	→	$OH + O_2^-$	$5.3 \cdot 10^9$	[62]
83	$O + OH^-$	→	HO_2^-	$4.2 \cdot 10^8$	[62]
84	$OH + Cl^-$	→	$ClOH^-$	$4.3 \cdot 10^9$	[57]
85	$OH + HClO$	→	$ClO + H_2O$	$9 \cdot 10^9$	[57]
86	$OH + ClO_2^- + H^+$	→	$ClO_2 + H_2O$	$6.3 \cdot 10^9$	[57]
87	$e_h^- + Cl$	→	Cl^-	$1 \cdot 10^{10}$	[57]
88	$e_h^- + Cl_2^-$	→	$2\ Cl^-$	$1 \cdot 10^{10}$	[57]
89	$e_h^- + ClOH^-$	→	$Cl^- + OH^-$	$1 \cdot 10^{10}$	[57]
90	$e_h^- + HClO$	→	$ClOH^-$	$5.3 \cdot 10^{10}$	[57]
91	$e_h^- + Cl_2$	→	Cl_2^-	$1 \cdot 10^{10}$	[57]
92	$e_h^- + Cl_3^-$	→	$Cl_2^- + Cl^-$	$1 \cdot 10^{10}$	[57]
93	$e_h^- + ClO_2^- + H^+$	→	$ClO + OH^-$	$4.5 \cdot 10^{10}$	[57]
94	$e_h^- + ClO_3^- + H^+$	→	$ClO_2 + OH^-$	$1 \cdot 10^{10}$	[63]
95	$H + Cl$	→	$Cl^- + H^+$	$1 \cdot 10^{10}$	[57]
96	$H + Cl_2^-$	→	$2\ Cl^- + H^+$	$8 \cdot 10^9$	[57]
97	$H + ClOH^-$	→	$Cl^- + H_2O$	$1 \cdot 10^{10}$	[57]
98	$H + Cl_2$	→	$Cl_2^- + H^+$	$7 \cdot 10^9$	[57]
99	$H + HClO$	→	$ClOH^- + H^+$	$1 \cdot 10^{10}$	[57]
100	$H + Cl_3^-$	→	$Cl_2^- + Cl^- + H^+$	$1 \cdot 10^{10}$	[57]
101	$HO_2 + Cl_2^-$	→	$Cl^- + HCl + O_2$	$4 \cdot 10^9$	[57]
102	HCl	→	$Cl^- + H^+$	$5 \cdot 10^5$	[57]

103	$Cl^- + H^+$	→	HCl	$6.29 \cdot 10^{-1}$	Calculated based on reaction 102 and $pK_a(HCl)$ = -5.9[64]
104	$HO_2 + Cl_2$	→	$Cl_2^- + O_2 + H^+$	$1 \cdot 10^{9}$	[57]
105	$HO_2 + Cl_3^-$	→	$Cl_2^- + HCl + O_2$	$1 \cdot 10^{9}$	[57]
106	$O_2^- + Cl_2^-$	→	$2\ Cl^- + O_2$	$1.2 \cdot 10^{10}$	[57]
107	$O_2^- + HClO$	→	$ClOH^- + O_2$	$7.5 \cdot 10^{6}$	[57]
108	$H_2O_2 + Cl_2^-$	→	$2\ HCl + O_2^-$	$1.4 \cdot 10^{5}$	[57]
109	$H_2O_2 + Cl_2$	→	$HO_2 + Cl_2^- + H^+$	$1.9 \cdot 10^{2}$	[57]
110	$H_2O_2 + HClO$	→	$HCl + H_2O + O_2$	$1.7 \cdot 10^{5}$	[57]
111	$OH^- + Cl_2^-$	→	$ClOH^- + Cl^-$	$7.3 \cdot 10^{6}$	[57]
112	$OH^- + Cl_2$	→	$HClO + Cl^-$	$6 \cdot 10^{8}$	[57]
113	$H^+ + ClOH^-$	→	$Cl + H_2O$	$2.1 \cdot 10^{10}$	[57]
114	$H_2O + Cl_2O_2$	→	$HClO + ClO_2^- + H^+$	$1 \cdot 10^{4}$	[57]
115	$H_2O + Cl_2O$	→	$2\ HClO$	$1 \cdot 10^{2}$	[57]
116	$H_2O + Cl_2O_4$	→	$ClO_2^- + ClO_3^- + 2\ H^+$	$1 \cdot 10^{2}$	[57]
117	$H_2O + Cl_2O_4$	→	$HClO + HCl + O_4$	$1 \cdot 10^{2}$	[57]
118	O_4	→	$2\ O_2$	$1 \cdot 10^{5}$	[57]
119	$Cl^- + Cl$	→	Cl_2^-	$2.1 \cdot 10^{10}$	[57]
120	$Cl^- + ClOH^-$	→	$Cl_2^- + OH^-$	$9 \cdot 10^{4}$	[57]
121	$Cl^- + HClO$	→	$Cl_2 + OH^-$	$1 \cdot 10^{1}$	[57]
122	$Cl^- + Cl_2$	→	Cl_3^-	$1 \cdot 10^{4}$	[57]
123	$ClOH^-$	→	$OH + Cl^-$	$6.1 \cdot 10^{9}$	[57]
124	Cl_2^-	→	$Cl + Cl^-$	$1.1 \cdot 10^{5}$	[57]
125	$2\ Cl_2^-$	→	$Cl_3^- + Cl^-$	$7 \cdot 10^{9}$	[57]
126	Cl_3^-	→	$Cl_2 + Cl^-$	$5 \cdot 10^{4}$	[57]
127	$2\ ClO$	→	Cl_2O_2	$1.5 \cdot 10^{10}$	[57]
128	$2\ ClO_2$	→	Cl_2O_4	$1 \cdot 10^{2}$	[57]
129	$Cl_2O_2 + ClO_2^-$	→	$ClO_3^- + Cl_2O$	$1 \cdot 10^{2}$	[57]
130	$2\ HClO$	→	$Cl^- + ClO_2^- + 2\ H^+$	$6 \cdot 10^{-9}$	[57]
131	$ClO_2^- + HClO$	→	$Cl^- + ClO_3^- + H^+$	$9 \cdot 10^{-7}$	[57]
132	$2\ HClO$	→	$O_2 + 2\ HCl$	$3 \cdot 10^{-10}$	[57]
133	$HClO + Cl^- + H^+$	→	$Cl_2 + H_2O$	$9 \cdot 10^{3}$	[57]
134	$Cl_2 + H_2O$	→	$HClO + Cl^- + H^+$	$1.5 \cdot 10^{1}$	[57]
135	$Cl_2^- + H_2$	→	$H + HCl + Cl^-$	$4.3 \cdot 10^{5}$	[57]
136	$2\ Cl$	→	Cl_2	$8.8 \cdot 10^{7}$	[65]

137	$ClO_2 + O_3$	→	$O_2 + ClO_3$	$1.1 \cdot 10^3$	[56]
138	$ClO_2 + OH$	→	$ClO_3^- + H^+$	$4 \cdot 10^9$	[56]
139	$ClO_2 + O^-$	→	ClO_3^-	$2.7 \cdot 10^9$	[56]
140	$ClO_2 + O_3^-$	→	$O_2 + ClO_3^-$	$1.8 \cdot 10^5$	[56]
141	$ClO_2 + O_3^-$	→	$O_3 + ClO_2^-$	$1.8 \cdot 10^5$	[56]
142	$ClO_2^- + O_3$	→	$O_3^- + ClO_2$	$4 \cdot 10^6$	[56]
143	ClO_2	→	$O_2 + Cl$	$6.7 \cdot 10^9$	[66]
144	$HClO$	→	$H^+ + ClO^-$	$2 \cdot 10^3$	[58]
145	$H^+ + ClO^-$	→	$HClO$	$5 \cdot 10^{10}$	[58]
146	$HClO_2$	→	$H^+ + ClO_2^-$	$9.53 \cdot 10^8$	[58]
147	$H^+ + ClO_2^-$	→	$HClO_2$	$5 \cdot 10^{10}$	[58]
148	$Cl + O_3^-$	→	$Cl^- + O_3$	$1 \cdot 10^9$	[58]
149	$ClO + O_3^-$	→	$ClO^- + O_3$	$1 \cdot 10^9$	[58]
150	$Cl_2^- + ClO_2$	→	$Cl_2O_2 + Cl^-$	$1 \cdot 10^9$	[58]
151	$Cl + ClO_2$	→	Cl_2O_2	$1 \cdot 10^9$	[58]
152	$ClO + ClO_2^-$	→	$ClO^- + ClO_2$	$9.4 \cdot 10^8$	[58]
153	$ClO^- + O^- + H^+$	→	$ClO + OH^-$	$2.3 \cdot 10^8$	[58]
154	$Cl^- + H_2O_2$	→	$ClO^- + H_2O$	$1.8 \cdot 10^{-9}$	[58]
155	$Cl^- + H_2O_2 + H^+$	→	$HClO + H_2O$	$8.3 \cdot 10^{-7}$	[58]
156	$ClO^- + H_2O_2$	→	$Cl^- + O_2 + H_2O$	$3.4 \cdot 10^3$	[58]
157	$HClO + HO_2^-$	→	$Cl^- + O_2 + H_2O$	$4.4 \cdot 10^7$	[58]
158	$Cl_2 + HO_2^-$	→	$2\ Cl^- + O_2 + H^+$	$1.1 \cdot 10^8$	[58]
159	$Cl + H_2O_2$	→	$Cl^- + H^+ + HO_2$	$2 \cdot 10^9$	[58]
160	$Cl + HO_2$	→	$Cl^- + H^+ + O_2$	$3.1 \cdot 10^9$	[58]
161	$Cl + OH^-$	→	$ClOH^-$	$1.8 \cdot 10^{10}$	[58]
162	$ClO_2 + H_2O_2$	→	$ClO_2^- + H^+ + HO_2$	$4 \cdot 10^0$	[58]
163	$ClO_2 + HO_2^-$	→	$ClO_2^- + HO_2$	$1.3 \cdot 10^5$	[58]
164	$ClO_2 + HO_2$	→	$ClO_2^- + H^+ + O_2$	$1 \cdot 10^6$	[58]
165	$ClO_2 + O_2^-$	→	$ClO_2^- + O_2$	$3 \cdot 10^9$	[58]
166	$ClO_2^- + O_2^-$	→	$ClO^- + O^- + O_2$	$4 \cdot 10^1$	[58]
167	$ClO + ClO_2$	→	Cl_2O_3	$7.4 \cdot 10^9$	[58]
168	$ClO + ClO_3$	→	Cl_2O_4	$7.4 \cdot 10^9$	[58]
169	$Cl_2O_2 + OH^-$	→	$Cl^- + ClO_3^- + H^+$	$1 \cdot 10^{10}$	[58]
170	$Cl_2O_3 + H_2O$	→	$HClO + ClO_3^- + H^+$	$1 \cdot 10^4$	[58]
171	$ClOH^-$	→	$Cl + OH^-$	$2.3 \cdot 10^1$	[58]
172	$Cl + H_2O$	→	$ClOH^- + H^+$	$1.8 \cdot 10^5$	[58]
173	$Cl_2^- + O_3$	→	$ClO + Cl^- + O_2$	$9 \cdot 10^7$	[58]

174	$Au(I)Cl_2^-$ + 2 Cl^- + OH	→	$Au(II)Cl_4^{2-}$ + OH^-	$2.6 \cdot 10^9$	[59]
175	$Au(III)Cl_4^-$ + H	→	$Au(II)Cl_4^{2-}$ + H^+	$5.7 \cdot 10^9$	[59]
176	$Au(III)Cl_4^-$ + e_h^-	→	$Au(II)Cl_4^{2-}$	$5.7 \cdot 10^9$	[59]
177	2 $Au(II)Cl_4^{2-}$	→	$Au_2Cl_6^{2-}$ + 2 Cl^-	$1.45 \cdot 10^8$	[54]
178	$Au(I)Cl_2^-$ + e_h^-	→	Au + 2 Cl^-	$8 \cdot 10^9$	[38]
179	$Au(I)Cl_2^-$ + H	→	Au + H^+ + 2 Cl^-	$8 \cdot 10^9$	[38]
180	$Au(I)Cl_2^-$ + HO_2^-	→	Au + HO_2 + 2 Cl^-	$1.89 \cdot 10^0$	[38]
181	$Au(I)Cl_2^-$ + H_2	→	Au + H^+ + H + 2 Cl^-	$7.4 \cdot 10^{-3}$	[38]
182	$Au(I)Cl_2^-$ + O_2^-	→	Au + O_2 + 2 Cl^-	$1.89 \cdot 10^0$	[38]
183	Au + $ClOH^-$ + Cl^-	→	$Au(I)Cl_2^-$ + OH^-	$1.83 \cdot 10^9$	assumed based on Ref.[38]
184	$Au_2Cl_6^{2-}$	→	$Au(I)Cl_2^-$ + $Au(III)Cl_4^-$	$1 \cdot 10^2$	[54]
185	$Au(III)Cl_4^-$ + Au + 2 Cl^-	→	$Au(II)Cl_4^{2-}$ + $Au(I)Cl_2^-$	0	[54]

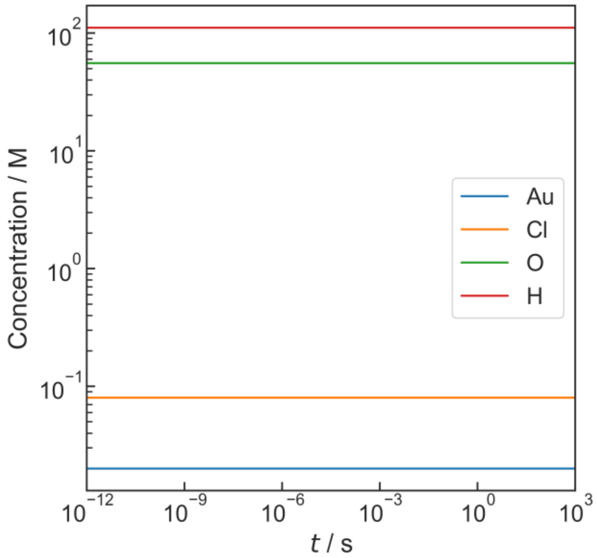

Figure S6: The concentrations of the elements remain constant, proofing the law of mass conservation during simulation.

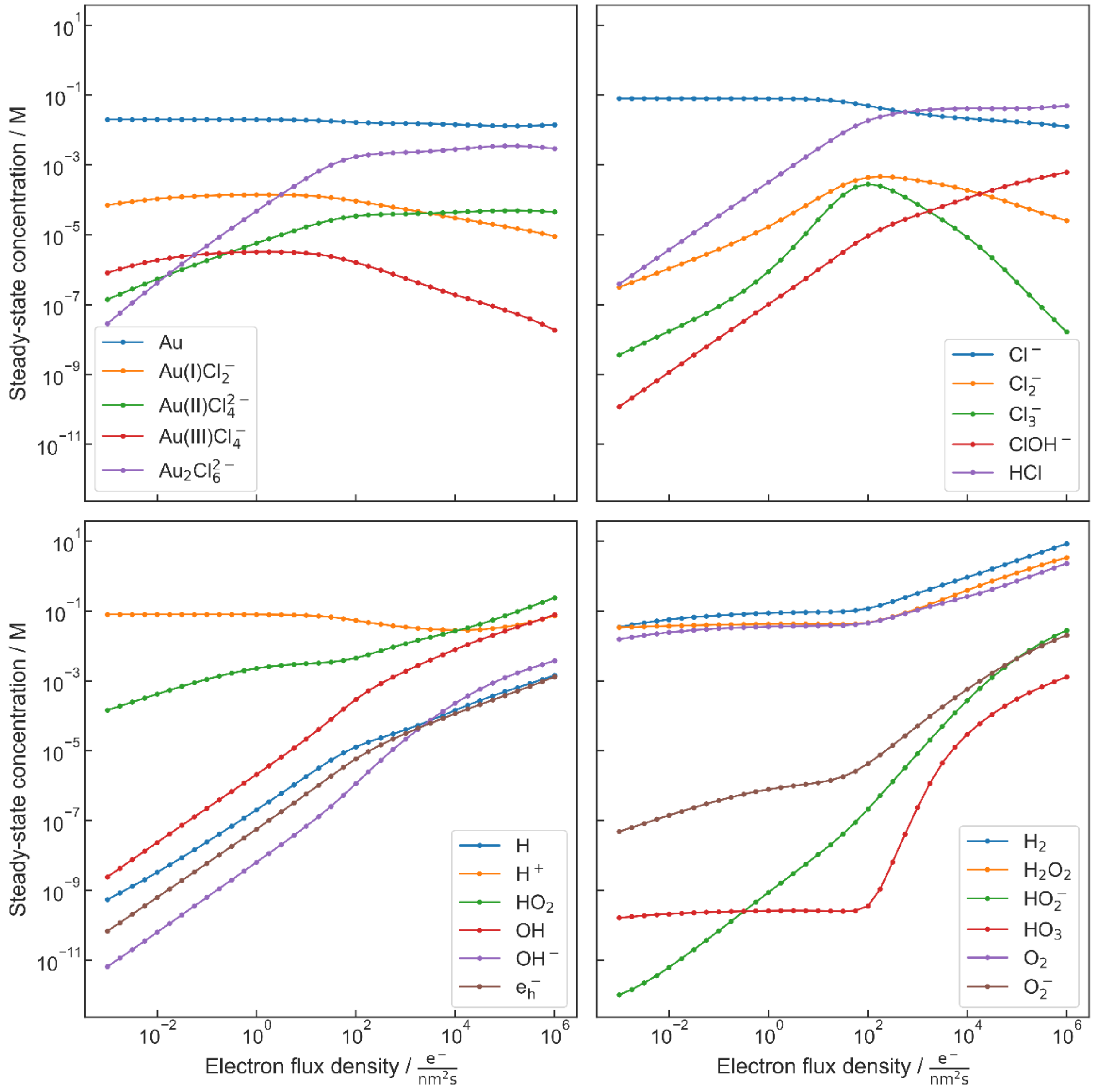

Figure S7: Logarithmic representation of steady state concentration of selected species exceeding at least 1% of the initial $HAuCl_4$ concentration of 20 mM versus electron-flux density.

S2 Exemplary growth of sub-threshold gold structures

S2.1. LP-TEM example

Figure S8(a) displays a gold nanostructure growth at "sub-threshold" conditions. The process can be partitioned into four phases:

First, a rapid agglomeration of (mainly) rod-shaped gold nanoparticles is observed within the first few frames of the experiment. The particles are already present prior to irradiation, which is discussed below. Second, rapid anisotropic growth in the direction of the long axis of the primary particles occurs. The growth rate in terms of projected area per time t in this phase

amounts to (100 ± 3) e⁻ (nm²s)⁻¹ (see orange line in ***Figure S8***(b)). This is comparable to the findings obtained by Park *et al.*[37] at 4000 e⁻ (nm²s)⁻¹. There, however, a strong shape-dependence of the growth rate is reported, which could explain the rapid growth observed here.

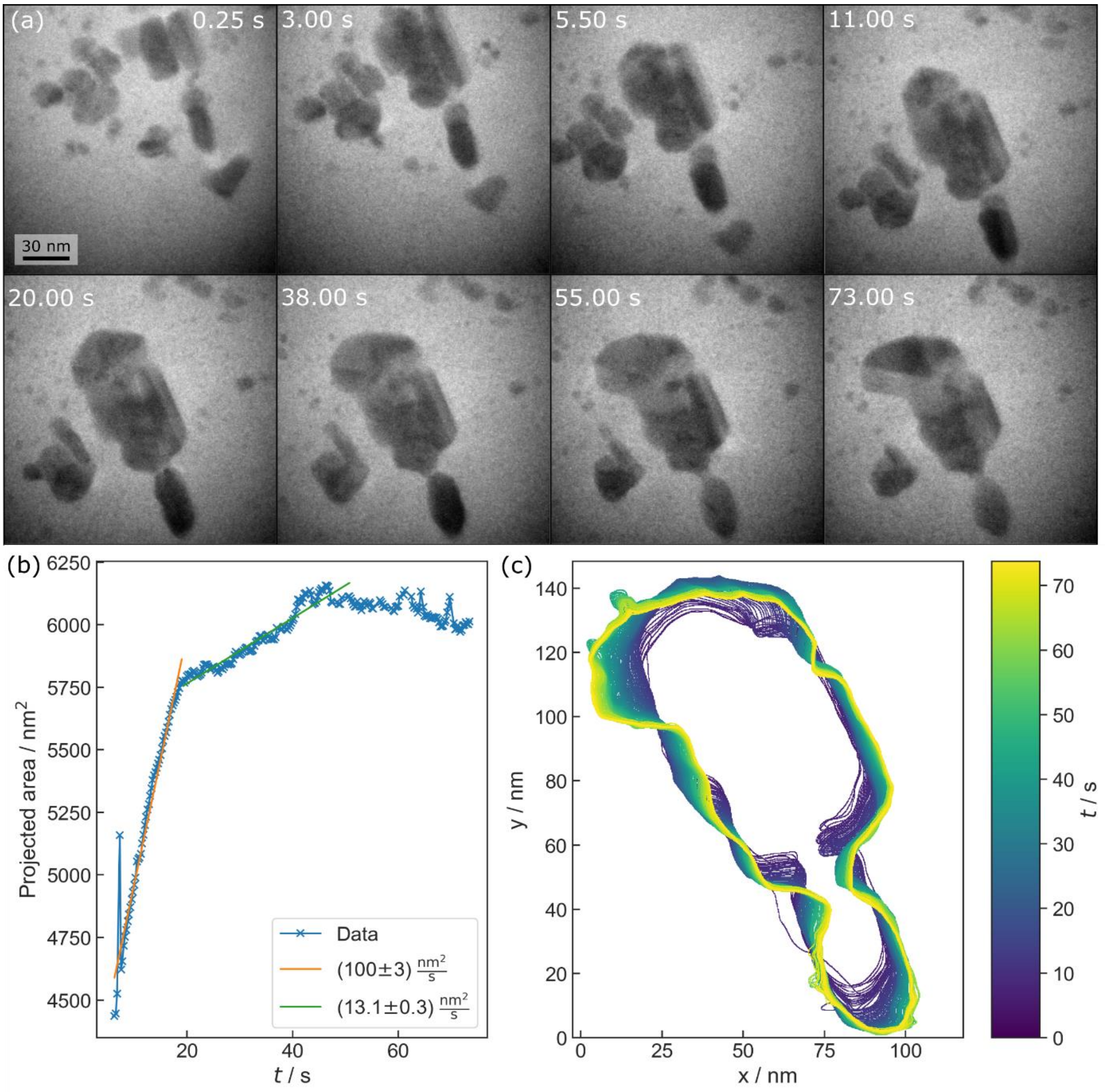

Figure S8: (a) Bright-field TEM micrographs showing coalescence and growth of a gold nanostructure in 20 mM $HAuCl_4$-solution at 540 e⁻(nm²s)⁻¹. (b) The evolution of the projected area of the main structure in (a) is displayed, indicating two regimes with different growth rates (see legend). The respective contour plots are depicted in (c), showing a change in growth direction.

Third, a substantially slower gold growth is evident (13.1±0.3) e⁻ (nm²s)⁻¹, green line in ***Figure S8***(b), which is accompanied by a change in the growth direction of about 120°, which fits well to the sixfold fcc symmetry of gold nanoparticles. Growth and reshaping are illustrated

in ***Figure S8***(c). Last, after about 52 s, the projected area remains approximately constant, while the crystal structure undergoes slight morphology changes only. The decrease in growth rate suggests that the local concentration gradients are seized so that the amount of solid gold remains in a dynamic equilibrium with the surrounding solution.

Low electron flux density-mediated nucleation has been observed repetitively, as shown below:

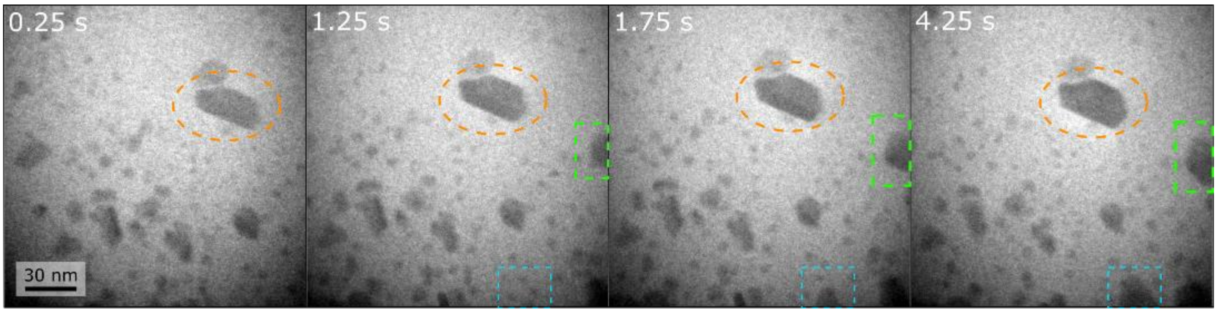

Figure S9: Bright-field TEM image series showing gold nanoparticle growth at 540 e-/(nm²s).

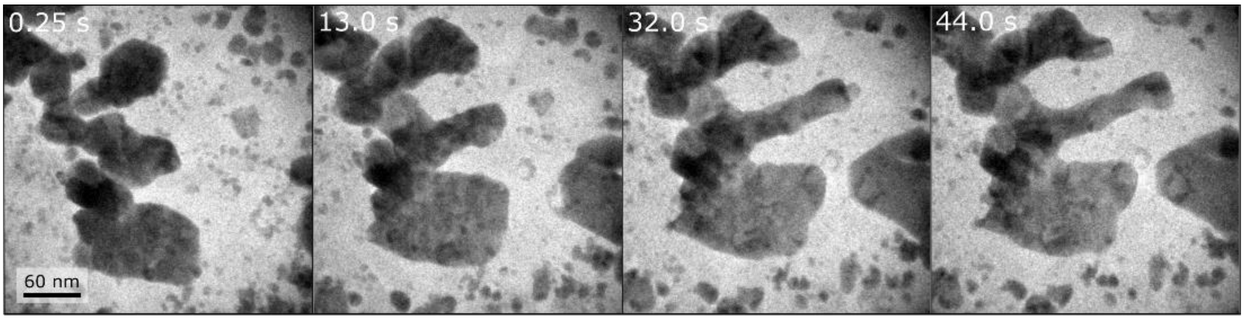

Figure S10: Bright-field TEM image series showing gold nanoparticle growth at 4.74 e^-/(nm²s).

S2.2. Additional data on X-ray induced gold precipitation

Note that the anisotropic particle distribution is due to the X-ray beam was not irradiating the whole sample area. This is caused by an anisotropic beam-shaping aperture that is installed prior to the liquid cell. Additionally, diffusion could contribute to the asymmetric shape of the area where precipitates are visible.

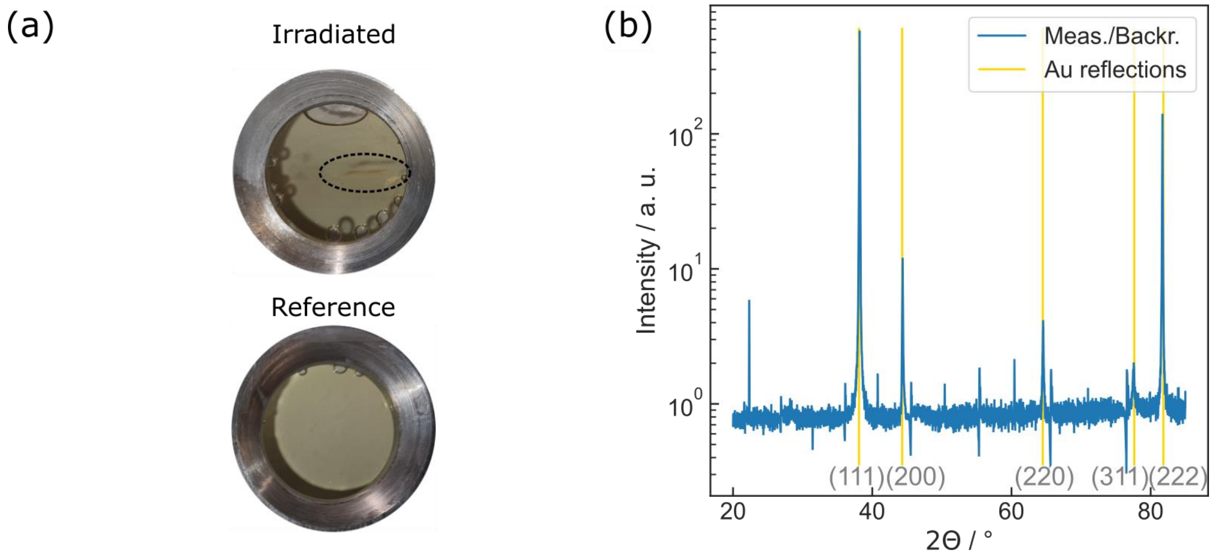

Figure S11: X-ray irradiation of a 20 mM HAuCl4-solution for 66 h at a dose rate of 1 Gy s^{-1}. (a) Gold-colored precipitations on the silica window of the liquid cell membrane are visible, which does not appear on a non-irradiated reference sample. (b) An XRD scan shows a clear sign of reflections matching well with the expected values for gold crystallites. The spikes jumping from extra-low to higher intensities stem from the background correction that mitigates the mica window's prominent contribution.

S3 Gold nanoparticle being etched in the vicinity of gas bubbles

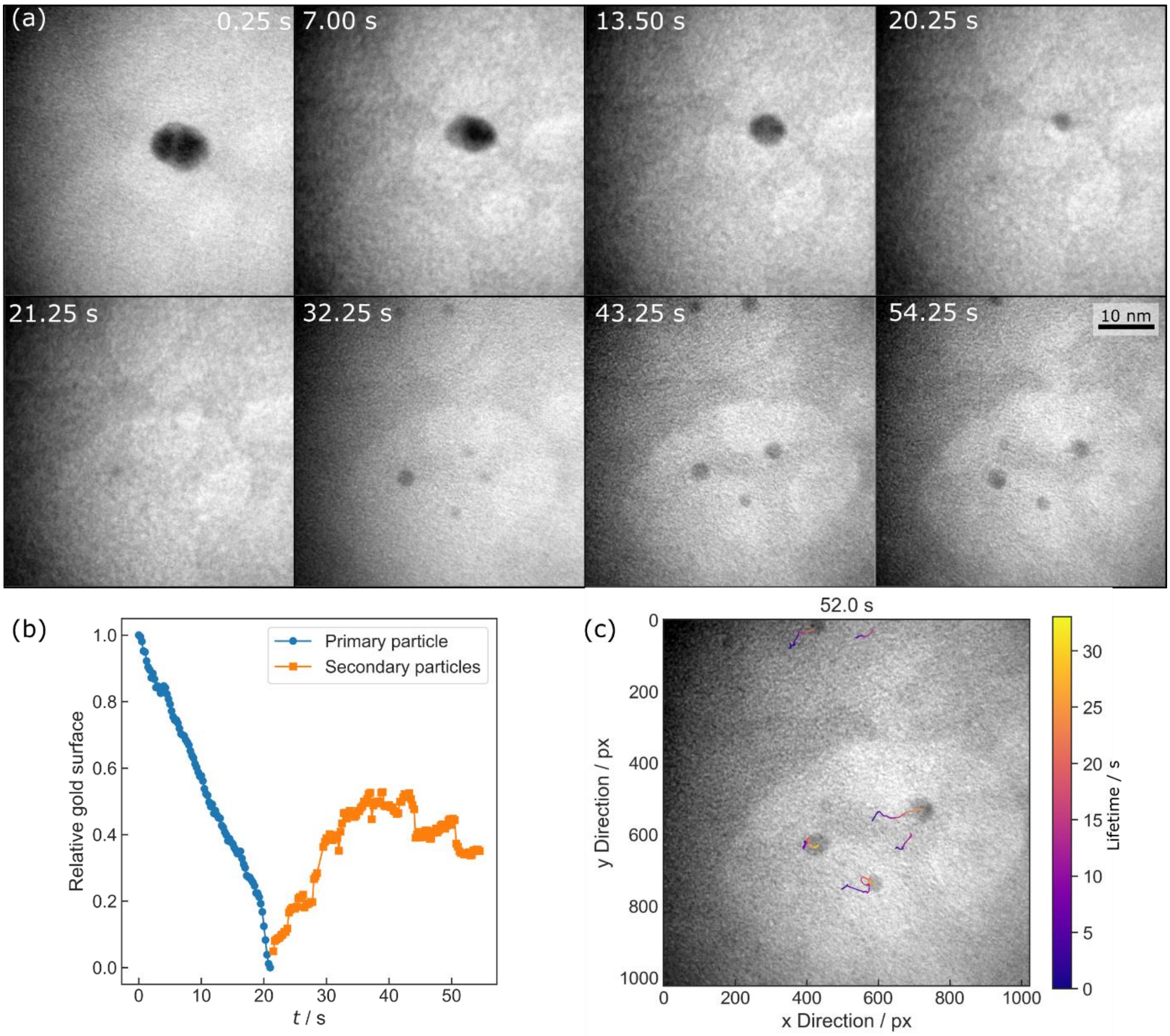

Figure S12: (a) Etching of a gold nanoparticle in 10 mM $HAuCl_4$-solution in the vicinity of gas bubbles and subsequent growth of nanoparticles at $3.7 \cdot 10^5$ $e^-(nm^2s)^{-1}$. (b) Relative gold surface evolution. (c) Trajectories of the secondary nanoparticles.

S4 Origin of the primary gold particles

During our experiments performed in GSMLCs, gold particles have been present already at first sight, even when broadcasting through the wells. This is remarkable, because no particles adhered to the graphene membranes without irradiation, as investigated by control experiments. The reduction of gold was verified *via* HRTEM measurement of the lattice plane distances using diffractograms (***Figure S13***).

Moreover, we did not observe a comparable experiment in liquid cells comprising two silicon nitride membranes, such as static liquid cells and flow cells. Here, only pattern formation and

particle growth in direct vicinity to the irradiated area was observed within minutes, as reported earlier.[92]

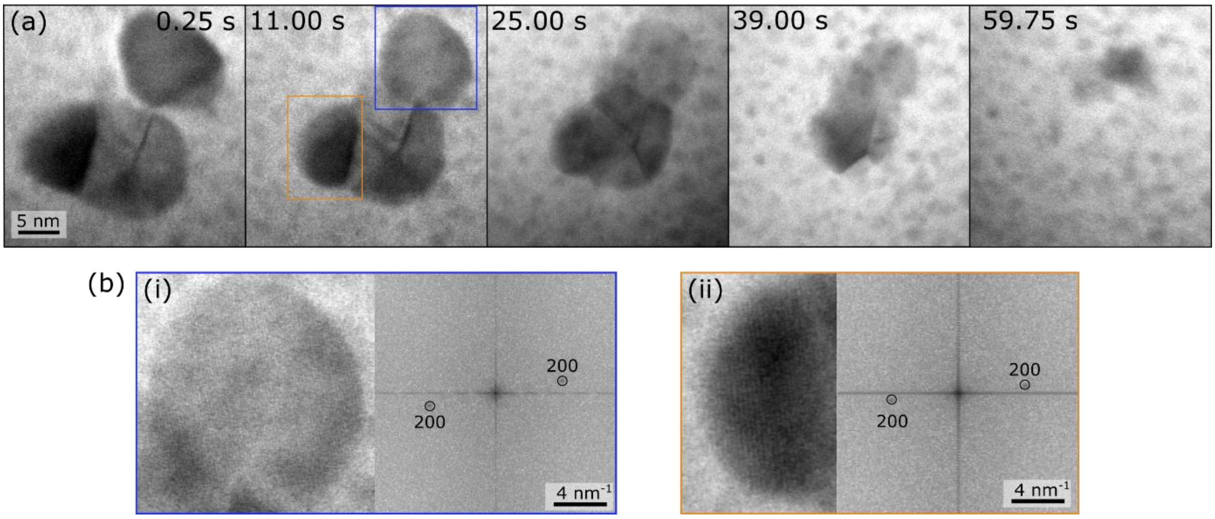

Figure S13:(a) Micrographs of LP-TEM observation of oxidative etching of a sixfold-twinned gold nanostructure. (b) and (c) show HRTEM data and corresponding FFTs. The reflexes fit well to {200} lattice planes of gold.

Thus, we hypothesize that the presence of the gold nanoparticles is related to the graphene membrane. Negative charging of thin carbon-based membranes during TEM has been reported, in particular of amorphous carbon (*a*C)[89,90] which can transfer onto GSMLCs during sealing.[69] In addition with the enhanced electrical conductivity of graphene compared to *a*C,[91] this could lead to a charge dissipation into regions not yet investigated during LP-TEM, spreading possible reduction sites even across different microwells. Moreover, this theory offers a possible explanation towards the mechanisms behind apparent mitigation of radiolysis and beam damage in graphene-based liquid cells.[26,27]

To test our theory, we mimicked possible electron beam-induced charging by conducting galvanostatic cathodic polarizations of different currents and durations applied in 20 mM $HAuCl_4$ solution. TEM grids covered with graphene were harnessed as the working electrode. The grids were prepared identical to those used for GSMLC-sealing. A standard three-electrode system was used with a leakless Ag/AgCl electrode (ET072-1, eDAQ Pty Ltd.) as reference

electrode and a Pt sheet as a counter electrode. A Zahner Zennium Electrochemical Workstation was employed for electrochemical tests.

At a low cathodic current of 200 nA, nanoparticles have been forming on the sample already after 5 min (***Figure S14***(a)). HRTEM investigations (***Figure S14***(b)) revealed *i. a.* multiple twinned structures matching well to primary particles etched *in situ* during LP-TEM (***Figure S13***(a)). Again, the diffractogram provides a strong indication of gold present, showing the reflexes matching well to {200} Au.[72]

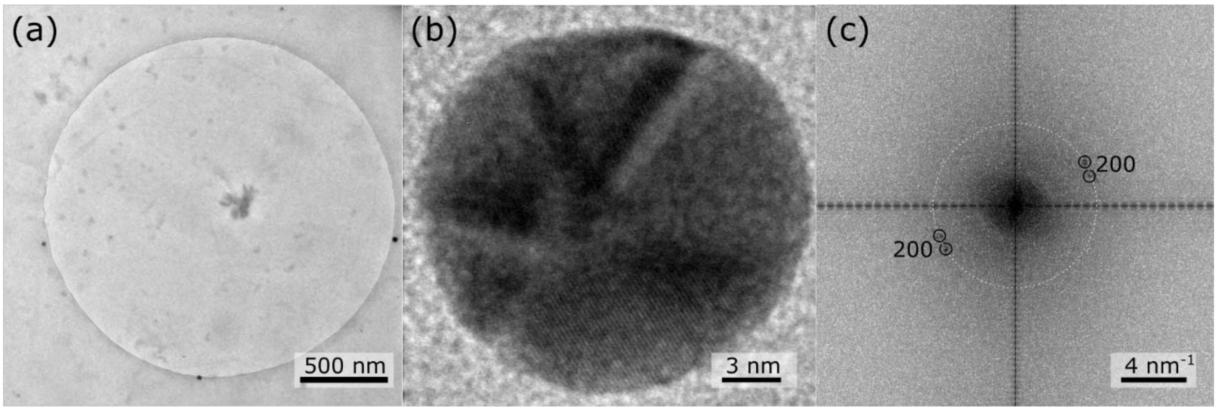

Figure S14: *Ex situ* TEM images of electrochemically grown gold nanostructures on freestanding few-layer graphene (a). (b) HRTEM image and (c) corresponding FFT of a fivefold-twinned gold nanoparticle.

By applying the same cathodic currents to pristine GSMLC carrier frames, reference experiments were performed on silicon nitride windows. Afterwards, no particles were observed *via* TEM on the free-standing membranes. This is, however, not surprising due to the insulating nature of silicon nitride.

S5 Relative Au^0 tracking for quantitative experimental descriptions

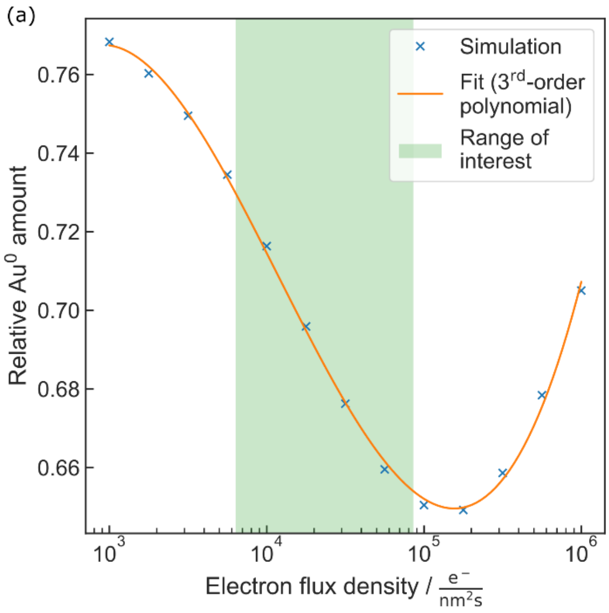

Figure S15: Fitting of a polynomial function on the range of interest for the experiment shown in ***Figure 6***(d).

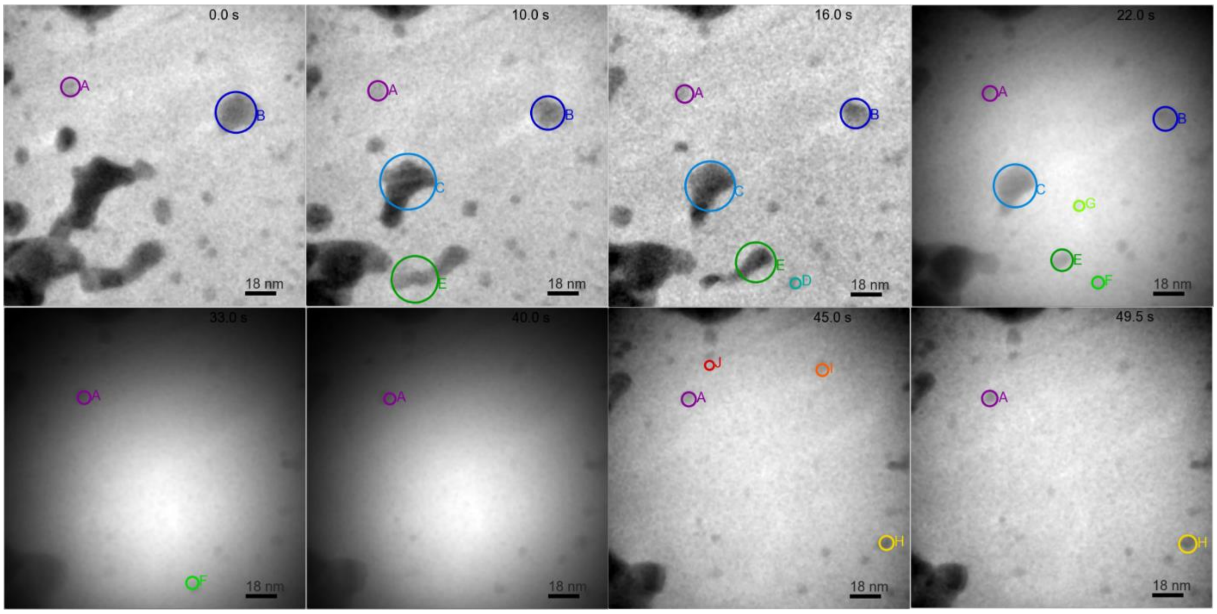

Figure S16: Bright-field TEM image series showing gold particle evolution in 20 mM $HAuCl_4$ evolution under dose rate change by beam contraction similarly to ***Figure 6***(a).

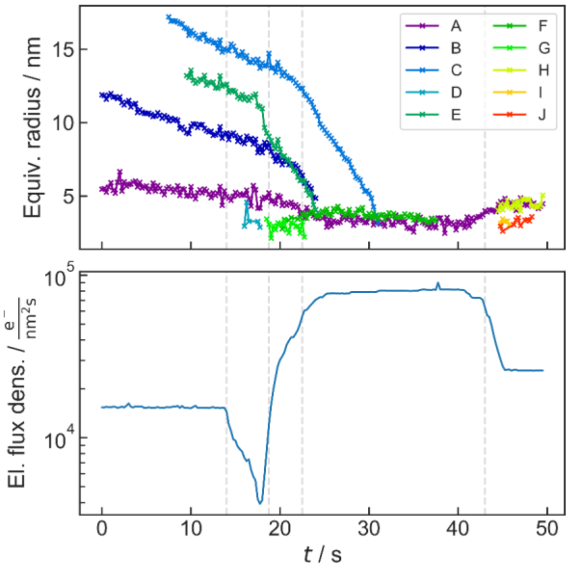

Figure S17: Equivalent radius evolution (top) and corresponding electron flux density (bottom) of the experiment sketched above (***Figure S16***). Dashed lines denote inflection points in the electron-flux density.

Assuming a spherical particle geometry to estimate the surface of the particle *A* is only a coarse approximation (c.f. the particle in ***Figure 6***(a)) of the true surface area *A'*. If shape evolutions are occurring isotropically, the related misfit of the model is correctable with a constant shape-transformation factor *s*. Hence, by regarding only changes of isotropic nature relative to an initial surface A_0 this factor is cancelled out and the spherical approximation remains applicable.

$$\frac{A'}{{A_0}'} = \frac{s \cdot A}{s \cdot A_0} = \frac{A}{A_0} \tag{S4}$$

S6 Dynamic steady-state

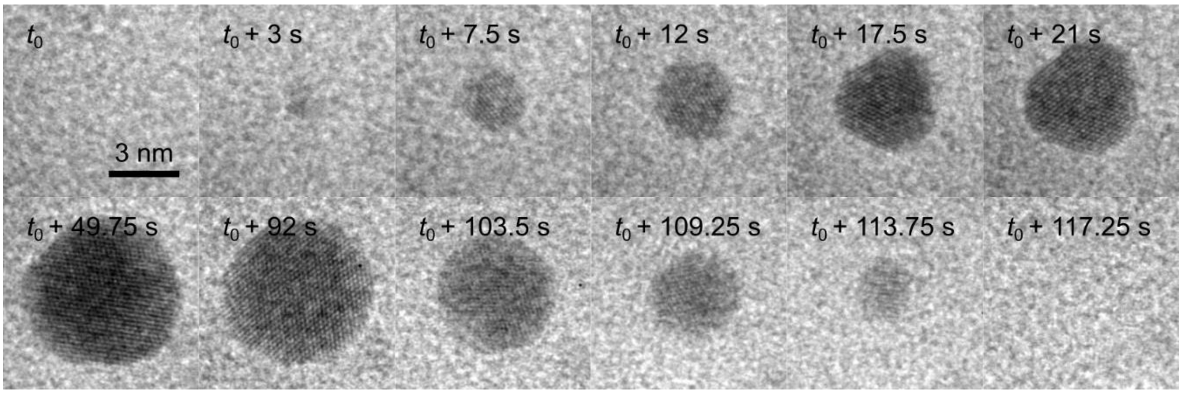

Figure S18: Fourier-bandpass filtered series of HRTEM micrographs of steady-state-mediated growth and dissolution of a gold nanoparticle in 1 mM $HAuCl_4$ solution irradiated with $4.5 \cdot 10^5$ e^- $(nm^2 s)^{-1}$.

S7 Concentration-dependent nanoparticle dissolution

The reduction of mass m of a nanoparticle over time t can be described *via* the Noyes-Whitney equation, with respect to the diffusion coefficient of the solvated species D, the surface area of the particle A, the liquid thickness of the boundary layer surrounding the particle L, the solubility of species c_s, and the equilibrium concentration of the dissolved species c_∞:[95]

$$\frac{dm}{dt} = \frac{DA}{L}(c_s - c_\infty) \qquad (S5)$$

Assuming a spherical particle with density ρ surrounded by a highly super-saturated solution allows reshaping of (S5) by expressing the mass in terms of density and radius and assuming c_∞ being small compared to c_s:[31,95]

$$\frac{dr}{dt} = \frac{Dc_s}{L\rho} \qquad (S6)$$

D/L matches the mass transfer coefficient K_{mass} [96]:

$$\frac{dr}{dt} = \frac{K_{mass} c_s}{\rho} \qquad (S7)$$

The Ostwald-Freundlich equation relates c_s to the corresponding bulk value c_0 *via* the ratio of critical radius r_{crit} to the actual radius r:[31]

$$c_s = c_0 e^{\frac{r_{\text{crit}}}{r}} \qquad (S8)$$

This allows for expressing the reduction of radius *r* over time as an ODE:

$$\frac{dr}{dt} = \frac{K_{\text{mass}} c_0}{\rho} e^{\frac{r_{\text{crit}}}{r}} = B e^{\frac{r_{\text{crit}}}{r}} \qquad (S9)$$

Equation (S9) can be fitted using a least-squares optimization of the parameters used for solving the ODE. In particular, this denotes the empirical pre-factor *B*, the boundary condition of *r* at the beginning of observation ($r_{t=0}$) and the desired quantity r_{crit}. This is exemplarily shown in ***Figure S19*** for the primary particle in ***Figure S12***.

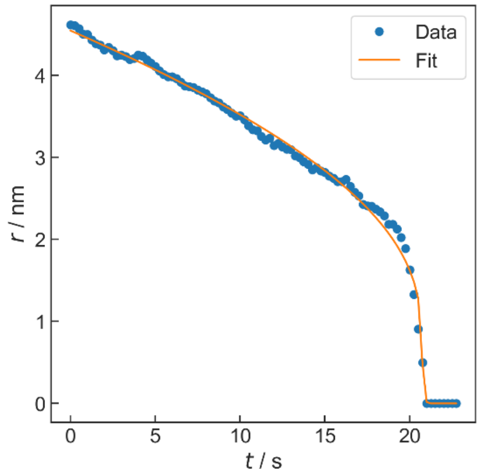

Figure S19: Exemplary fit of the critical radius on the particle shown in ***Figure S12*** corresponding to the highest $R^2_{\text{adj.}}$ in ***Figure S20***.

Consequently, the numerical ODE solver is connected in series with the least square optimization algorithm. As both tools converge against (possible) local minima, the goodness of the outcome (quantified by the adjusted coefficient of determination $R^2_{\text{adj.}}$) is highly sensitive to the starting parameters. Therefore, those were varied systematically to detect the global minimum within this parameter space (***Figure S20***).

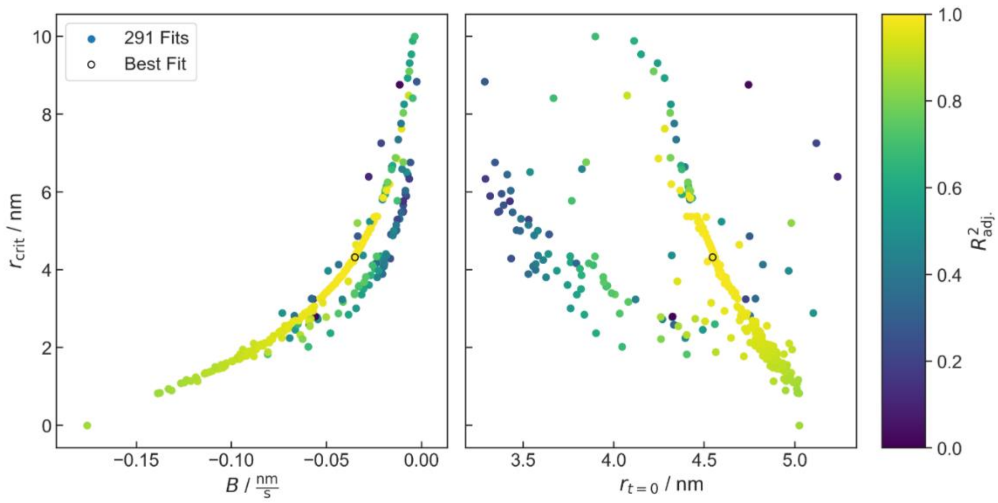

Figure S20: Exemplary parameter space in critical radius fitting. The colorbar illustrates the adjusted coefficient of determination $R^2_{adj.}$.

S8 Chemical potential calculation

In the following, the transition from a purely solid gold phase into partially dissolved gold in steady state condition with the residual solid phase after infinite time is discussed:

$$\mathrm{Au_{solid}} \rightarrow \mathrm{Au_{solid}} + \mathrm{Au_{solvated}} \tag{S10}$$

Mass conservation demands that the total amount of Au remains constant:

$$\mathrm{Au_{solid}}(t=0) = \mathrm{Au_{solid}}(t=\infty) + \mathrm{Au_{solvated}}(t=\infty) \tag{S11}$$

As discussed above, stray irradiation, irradiation during alignment, and graphene-modulated charge-dissipation reduced a major amount of the gold initially stored in $HAuCl_4$ to Au^0. As the volume remains unchanged, this can be directly expressed by concentrations c, where c_0 is the initially solvated amount.

- $c_0(\mathrm{HAuCl_4}) \approx c(\mathrm{Au_{solid}}(t=0)) = c(\mathrm{Au_{solid}}(t=\infty)) + c(\mathrm{Au_{solvated}}(t=\infty))$ (S12)

As shown above, the system converges towards a steady state. Thus, $t=\infty$ can be identifyed with the steady-state condition.

$$c_0(\mathrm{HAuCl_4}) = c_{\text{steady state}}(\mathrm{Au_{solid}}) + c_{\text{steady state}}(\mathrm{Au_{solvated}}) \qquad (S13)$$

As Au is insoluble in water, $c_{\text{steady state}}(\mathrm{Au_{solid}})$ is assumed to equal the simulated concentration of Au^0.

If the transition does reach a steady state situation, the net concentration in the phases does not change anymore. Thus, the chemical potentials of these phases must be equalized. Therefore, describing any of the final phases is sufficient for calculating the chemical potential difference $\Delta\mu$. At a given thermal energy $k_\mathrm{B}T$, $\Delta\mu$ of the transition shown in (S10) is characterized by the fraction of solid and solvated amount of gold prior and after the transition, x_0 and x_∞.[93,94]

$$\Delta\mu = k_B T \;\, ln\left(\frac{x_0}{x_\infty}\right) \qquad (S14)$$

Accounting for the description of the two situations above yields the following expressions for x_0 and x_∞:

$$x_0 = \frac{c(\mathrm{Au_{solid}}(t=0))}{c_0(\mathrm{HAuCl_4})} = 1 \qquad (S15)$$

$$x_\infty = \frac{c_{\text{steady state}}(\mathrm{Au^0})}{c_0(\mathrm{HAuCl_4})} \qquad (S16)$$

Inserting (S15) and (S16) in (S14) yields the desired expression shown in the main manuscript:

$$\Delta\mu = k_\mathrm{B}T \ln\left(\frac{1}{\frac{c_{\text{steady state}}(\mathrm{Au^0})}{c_0(\mathrm{HAuCl_4})}}\right) = k_\mathrm{B}T \ln\left(\frac{c_0(\mathrm{HAuCl_4})}{c_{\text{steady state}}(\mathrm{Au^0})}\right) \qquad (S17)$$

S9 Supporting video captions

Video S1: *In situ* LP-TEM video showing gold nanoparticle dynamics in 20 mм $HAuCl_4$ under repetitive dose-rate change. Speed is 5-fold accelerated. A scale bar can be found in ***Figure 6*** in the main manuscript.

Video S2: *In situ* LP-TEM video showing gold nanoparticle dynamics in 20 mм aqueous $HAuCl_4$ solution at low dose rates. Speed is 5-fold accelerated. A scale bar can be found in Supporting ***Figure S8*** in the supplementary information.

Video S3: *In situ* LP-TEM video showing gold nanoparticle dynamics in 10 mм aqueous $HAuCl_4$ solution at a solid-liquid-gas interface. Speed is 5-fold accelerated. A scale bar can be found in Supporting ***Figure S10*** in the Supporting Information.

Video S4: Sharpened *in situ* LP-TEM video showing gold nanoparticle dynamics in 20 mм aqueous $HAuCl_4$ solution at high resolution. Speed is 5-fold accelerated. A scale bar can be found in Supporting ***Figure S11*** in the Supporting Information.

Video S5: Sharpened *in situ* LP-TEM video showing gold nanoparticle dynamics in 1 mм aqueous $HAuCl_4$ solution at high resolution. Speed is 5-fold accelerated. Please refer to Supporting ***Figure S14*** in the Supporting Information for scale.
